# Supplementary material for: Allosteric regulation and crystallographic fragment screening of SARS-CoV-2 NSP15 endoribonuclease
Source: Nucleic Acids Res. 2023 Apr 28;51(10):5255–70. doi: 10.1093/nar/gkad314 (PMC10250223; doi:10.1093/nar/gkad314)
Supplement: gkad314_Supplemental_Files [file gkad314_supplemental_files.zip › Supplementary datasets.zip!/139/Refine_1/01-BUSTER/Cycle-4/LIST.html]

Refining

**BUSTER 2.X**: A suite of programs for macromolecular structure refinement by maximum-likelihood and Bayesian methods  
Copyright © 1992-2020 Global Phasing Ltd.  
All rights reserved.  
Authors: G. Bricogne, E. Blanc, M. Brandl, C. Flensburg, P. Keller, W. Paciorek, P. Roversi, O. Smart, C. Vonrhein, T. Womack  

Please reference use of the software as:

- BUSTER-TNT 2.X, Global Phasing Ltd, Sheraton House, Cambridge CB3 0AX, UK

Version: 2.10.3 for linux64-ifort made on May 20 2020 at 01:14:34  
Licenced to: Diamond Light Source, Scientific Software  

```
---


### Parsing of the BUSTER cardfile

TITLE : autoBUSTER refinement

   Cell information

   Symmetry information

 Using CCP4 X-ray form factors.

   Missing structure chemical composition

 Using ATOM block for chemical composition.


   Shell information

### Parsing of the TNT cardfile


---


### Reading observations file

 Number of reflexions in input:  82647

      Number of accepted reflexions:  82309

      Number of rejected reflexions:      0

   Reflexions in test set:            4081

   Reflexions in working set:        78228


---


### 1. Generating initial model

 2.10 * Shannon sampling rate. NX NY NZ:   360  360  240


  Effective Shannon rates are:    2.50    2.50    2.35


   Allocated BUFFER of size:    119.312 Mb.

   Initial Geometry Information


 No missing atoms declared


   Summary of model volumes

 No missing atoms declared: no prior computed


 Computing bulk solvent model scattering


 NCSMASK PDB radius for solvent mask:     2.15 Angstrom

 running PDBSET (setting cell and spacegroup P1) ... done
 running NCSMASK [1] (creating mask, SMOOTH 0.906025) ... done
 running MAPDUMP (to check if reduction to unit cell is needed) ... done
 running MAPMASK (converting mask to map) ... done

   Generation of babslv by theta-filter

   Electron density in frg mask:  0.420 Å-1

 FOBS  put on rough absolute scale by Wilson plot :
           K        =  0.63350E+02
           B        =     -7.443

 

### Cycle 0001: MaxLik scaling and refinement


   MaxLik Scaling Cycle 0001

   Wilson plot

   Statistics in resolution bins just after scaling
```

|  |  |
| --- | --- |
|  | - Rxpct factors plot- Rxpct factors table- <Log-Lik Gain>- Ln Sigma A- Correlation Coefficients |

```
   <Log-Likelihood> of null-hypothesis

Structure Factor Amplitudes


   Average Figure of Merit

 Switching off refinement of overall temperature factor


 Added B =     0.8541 Å2 to all atoms in fragment


 Added B =     0.8541 Å2 to B_SOLV

```
gelly refinement module
 Authors (2004-2009) O. S. Smart, C. Flensburg, W. Paciorek, C. Vonrhein, 
                     M. Brandl, T. O. Womack and G. Bricogne
         (2010-2011) O. S. Smart, C. Flensburg, W. Paciorek, C. Vonrhein, 
                     T. O. Womack and G. Bricogne
         (2012-2014) O. S. Smart, C. Flensburg, W. Paciorek, C. Vonrhein, 
                     and G. Bricogne
         (2015-2020) G. Bricogne, C. Flensburg, W. Paciorek, C. Vonrhein
 Copyright © 2004-2020 Global Phasing Ltd. 
 All rights reserved.
 gelly module compilation date May 20 2020 01:11:20
 
 gelly uses routines from TNT geometry. References: 
     Tronrud, D. E., Ten Eyck, L. F., Matthews, B. W. (1987)
     "An Efficient General-Purpose Least-Squares Refinement Program 
     for Macromolecular Structures". Acta Crystallogr A, 43:489-501.
     Tronrud, D. E. (1997). "The TNT Refinement Package", 
     in Macromolecular Crystallography, Part B, Eds Charlie Carter 
     and Robert Sweet, Methods in Enzymology, 277:306-319.
 wildcard matching function by Julian Robichaux http://www.nsftools.com

 No -random_seed flag found. Random number stream initialised using:
2147483562
2147483398

 Control flags set to:  -autoncs -hydrogens_input_occ2zero \
                     -hydrogens_output_occ2heavy -screen \
                     100 -screen_sigma \
                     3.0 -keeppdb \
                     1000000 -taomonitor_verbose \
                     2 
 

 ############### Starting setup via TNT routines

 Setting up tnt routines including license check:

                   TNT Stereochemistry Program
 Version: 5.22.3.0
 Licence for Diamond Light Source, Scientific Software is valid
 
 Using TNT geometry routines to load the tnt control file: gelly.tnt
 Using TNT routines to load initial coordinates from file fragment.cor
 Expecting 6119 atoms

 Found     2192 "NOTE BUSTER_RESIDUE_ATOM_DATA" cards
 Have copied information for    6119 atoms from TNT to gelly's store 
 
 The TNT WEIGHT cards used by this run:
 -----WEIGHT       BOND     2.0
 -----WEIGHT       ANGLE    2.0
 -----WEIGHT       IMPROPER 0.0
 -----WEIGHT       TORSION  2.0
 -----WEIGHT       PSEUDO   0.0
 -----WEIGHT       TRIGONAL 2.0
 -----WEIGHT       PLANE    5.0
 -----WEIGHT       CONTACT  5.0
 -----WEIGHT       BCORREL  10.0
 -----WEIGHT       CHIRAL   5.0
 -----WEIGHT       NCS      50.0
 -----WEIGHT       IDEAL    4.0
 -----WEIGHT       RFACTOR  5.44
 
 Dictionary versions picked up from NOTE BUSTER_RESTRAINT_DICT cards as: 
 ------ protgeo_eh99.dat (V1.6.4.17) 20191001  STANDARD AMINO ACID DICTIONARY. BONDS AND ANGLES FROM ENGH AND HUBER EH99. OTHER VALUES BASED ON PREVIOUS TNT OR TAKEN FROM CCP4. INCLUDES HYDROGEN ATOMS.
 ------ exoticaa.dat (V1.3.4.15) 20200327  COLLECTION OF NON-STANDARD AMINO ACIDS, MAINLY EH91 WITHOUT IDEAL DISTANCE INFO
 ------ nuclgeo.dat (V1.13.4.8) 20190909
 ------ bcorrel.dat (V1.15.10.2) 20190616
 ------ contact.dat (V1.15.12.10) 20191121
 ------ idealdist_contact.dat (V1.3.4.12) 20191001  IDEAL-DISTANCE CONTACT TERM DATA AS USED IN PROLSQ. VALUES USED HERE ARE BASED ON DATA HARVESTING OF PDB AS OF 20190109.
 ------ restraints for LIG (Unknown) from cif dictionary Z85934875.cif
 ------ restraints for CIT (CITRIC ACID) from cif dictionary CIT.cif; generated by GRADE_PDB_LIGAND 1.2.12 (October 4 2016) using MOGUL 1.7.2(RC1), CSD as538be, with quantum mechanics RM1 ((DatabaseID PDB-CIT))
 ------ assume.dat (V1.9.4.2) 20180705
 
 Have picked up spacegroup name for pdb output as 'P 63      '
 Setting up Chain/Residue arrays
 
 Have command line specification -autoncs*
     Have found       1 NCS relations
     Pairwise matching residue number and type between chains: 
                    A        B        S    
          A         .       99%       -   
          B                  .        -   
          S                           .   
       Table key--                                                       
          all   : two chains have 100% match and same number of residues 
         multi  : ditto and they contribute to a multi chain ncs cluster 
          ***%  : two chains have >80% matching residue number/type      
           -    : chains appear to be unrelated                          
      chain A     have identified ncs to     1 other chains. So will weight each autoncs by (       6.666/#)=      6.6660
      chain B     have identified ncs to     1 other chains. So will weight each autoncs by (       6.666/#)=      6.6660
      chain S     have identified ncs to     0 other chains. 
     For .Gelly cards produced see below - after pruning
 
 Setting up BUSTER_SET groups
 Have found    89 BUSTER_SET cards
 Now silently applying automatic/dictionary set definitions. For a verbose listing use '-verbose_set' flag.
 Applying user defined set definitions:
 =====NOTE BUSTER_SET ncsautoXcld = Water --> set 'ncsautoXcld' now has   581 atoms 
 =====NOTE BUSTER_SET ncsautoABset = Chain_B \ ncsautoXcld --> set 'ncsautoABset' now has  2770 atoms 
 Have defined    48 sets: 
      set:                 'All' has   6119 atoms in   1281 residues - automatically defined
      set:               'Empty' has      0 atoms in      0 residues - automatically defined
      set:            'ExoticAA' has      0 atoms in      0 residues - defined by dictionary file
      set:           'ExoticNUC' has      0 atoms in      0 residues - defined by dictionary file
      set:              'NotHET' has   5514 atoms in    698 residues - defined by dictionary file
      set:             'Chain_A' has   2768 atoms in    350 residues - automatically defined
      set:             'Chain_B' has   2770 atoms in    350 residues - automatically defined
      set:             'Chain_S' has    581 atoms in    581 residues - automatically defined
      set:               'Water' has    581 atoms in    581 residues - defined by dictionary file
      set:          'StdProtein' has   5514 atoms in    698 residues - defined by dictionary file
      set:             'Protein' has   5514 atoms in    698 residues - defined by dictionary file
      set:                'Back' has   2794 atoms in    698 residues - defined by dictionary file
      set:                'Side' has   2720 atoms in    650 residues - defined by dictionary file
      set:        'RnaCanonical' has      0 atoms in      0 residues - defined by dictionary file
      set:        'DnaCanonical' has      0 atoms in      0 residues - defined by dictionary file
      set:     'RnaDnaCanonical' has      0 atoms in      0 residues - defined by dictionary file
      set:     'DnaRnaCanonical' has      0 atoms in      0 residues - defined by dictionary file
      set:         'RnaExtended' has      0 atoms in      0 residues - defined by dictionary file
      set:         'DnaExtended' has      0 atoms in      0 residues - defined by dictionary file
      set:      'RnaDnaExtended' has      0 atoms in      0 residues - defined by dictionary file
      set:      'DnaRnaExtended' has      0 atoms in      0 residues - defined by dictionary file
      set:                 'Dna' has      0 atoms in      0 residues - defined by dictionary file
      set:                 'Rna' has      0 atoms in      0 residues - defined by dictionary file
      set:              'RnaDna' has      0 atoms in      0 residues - defined by dictionary file
      set:              'DnaRna' has      0 atoms in      0 residues - defined by dictionary file
      set:             'Nucleic' has      0 atoms in      0 residues - defined by dictionary file
      set:             'Polymer' has   5514 atoms in    698 residues - defined by dictionary file
      set: 'BufferWeichenberger' has     13 atoms in      1 residues - defined by dictionary file
      set:    'BufferAdditional' has      0 atoms in      0 residues - defined by dictionary file
      set:              'Buffer' has     13 atoms in      1 residues - defined by dictionary file
      set:         'MetalAlkali' has      0 atoms in      0 residues - defined by dictionary file
      set:  'MetalAlkalineEarth' has      0 atoms in      0 residues - defined by dictionary file
      set:     'MetalTransition' has      0 atoms in      0 residues - defined by dictionary file
      set: 'MetalPostTransition' has      0 atoms in      0 residues - defined by dictionary file
      set:    'MetalLanthanides' has      0 atoms in      0 residues - defined by dictionary file
      set:               'Metal' has      0 atoms in      0 residues - defined by dictionary file
      set:         'SugarAlphaD' has      0 atoms in      0 residues - defined by dictionary file
      set:         'SugarAlphaL' has      0 atoms in      0 residues - defined by dictionary file
      set:               'Sugar' has      0 atoms in      0 residues - defined by dictionary file
      set:               'Other' has     24 atoms in      2 residues - defined by dictionary file
      set:            'Hydrogen' has      0 atoms in      0 residues - defined by dictionary file
      set:         'nonHydrogen' has   6119 atoms in   1281 residues - defined by dictionary file
      set:             'OccZero' has      0 atoms in      0 residues - defined by dictionary file
      set:          'OccNonZero' has   6119 atoms in   1281 residues - defined by dictionary file
      set:             'OccFull' has   6076 atoms in   1278 residues - defined by dictionary file
      set:          'OccNonFull' has     43 atoms in      3 residues - defined by dictionary file
      set:         'ncsautoXcld' has    581 atoms in    581 residues - user specified
      set:        'ncsautoABset' has   2770 atoms in    350 residues - user specified
 No command line arguments '-occ_allhet' or '-occ_restyp' so will not setup automated occcupancy refinement
 
 Interpreting CONSTANT and FREE cards: 
 =====CONSTANT     OCC
 Have found and interpreted      1 CONSTANT cards in the TNT input. 
 Before any COMBINE - Number of atoms with fixed XYZ =     0
 Before any COMBINE - Number of atoms with fixed B   =     0
 Before any COMBINE - Number of atoms with fixed OCC =  6119
 Before any COMBINE - Number of atoms with fixed BIJ =   581
                            cf total number of atoms =  6119
 
 There are no COMBINE cards to be found in the TNT card input 
 
 There are no NOTE BUSTER_COMBINE_RES cards to be found in the TNT card input 

 ############### Working out full connectivity list


 ############### Working out equivalent atoms


 Residue with most atoms given below:
        A|58:N(TRP)         
        A|58:CA(TRP)        
        A|58:CB(TRP)        
        A|58:CG(TRP)        
        A|58:CD1(TRP)       
        A|58:NE1(TRP)       
        A|58:CE2(TRP)       
        A|58:CD2(TRP)       
        A|58:CE3(TRP)       
        A|58:CZ3(TRP)       
        A|58:CH2(TRP)       
        A|58:CZ2(TRP)       
        A|58:C(TRP)         
        A|58:O(TRP)         


 Have no '-equiv_check_plus' argument - so will take atom type into
 account when checking for equivalence of otherwise identical atoms.
 

 ############### Looking at dihedrals


 ############### Looking at chirals

 NOTE BUSTER_CHIRAL_ANGLECUT card found - read value=    -31.00 degs from it
 NOTE BUSTER_CHIRAL_SIGMA card found - read value=      1.70 degs from it
 RANGE allowed for B variables in refinement:   from      3.00000 to    300.00000

 ############### Preparing for PDB output


 ############### Processing extra restraints explicitly defined

 No 'NOTE BUSTER_TARGET' cards or -target?? args found (used to read coordinates files for soft ncs to external)
 No 'NOTE BUSTER_OCCSUM'     cards found - so not using sum of occupancy restraints
 No 'NOTE BUSTER_DISTANCE'   cards found - so not setting up any additional atom-atom distance restraints
 No 'NOTE BUSTER_UTILANGLE'  cards found - so not setting up any additional restraints on any angle
 No 'NOTE BUSTER_UTILTOR'    cards found - so not setting up any additional sinusoidal restraints on torsions
 
 --- Have some similarity definitions 
     Similarity restraints on RMSD use superposition routines from: 
     Coutsias, E.A., Seok, C., Dill, K.A.(2004)
     "Using quaternions to calculate RMSD", J. Comput. Chem., 25:1849-1857 

 Have found    1 'NOTE BUSTER_SIM_DEFINE' cards 
     Maximum number of chains involved in any SIM group (excluding the template)=     1


 ########## Interpreting card: NOTE BUSTER_SIM_DEFINE ncsautoAB ncsautoABset A
     Number of atoms in template set=  2770
     Number of related chains (other than template) in SIM set=    1
     List of chains to be restrained (excluding template)=   A   
     Pairwise RMSD, XYZ in Angstroms, for SIM group: ncsautoAB
                 B         A   
     B         -----     ----- 
     A         0.594     ----- 
     Overall RMSD,XYZ for the SIM group is     0.594 Angstroms
     Pairwise rms deviation of [individual B's minus their set/chain average] in Angs**2
                 B         A         Bavg
     B         -----     -----     52.78
     A         44.53     -----     88.92
     Overall RMSD,B   for the SIM group is    44.529 Angs**2
     The number of common atoms for the above fits:
                 B         A   
     B         -----     ----- 
     A          2757     ----- 
     (compared to the template set that has    2770 atoms in it)
     List of atoms that are absent in one or more chains for this group: 
                                    B    A   
                    C1      401     +    -
                    O1      401     +    -
                    O2      401     +    -
                    C2      401     +    -
                    C3      401     +    -
                    O7      401     +    -
                    C4      401     +    -
                    C5      401     +    -
                    O3      401     +    -
                    O4      401     +    -
                    C6      401     +    -
                    O5      401     +    -
                    O6      401     +    -
     (key  +  atom present for this chain 
           -  atom absent  for this chain)
 transforms for each of the superpositions orthogonal matrix r11 r12 r13 r21 r22 r23 r31 r32 r33 tx ty tz (PDBSET syntax): 
TRANS_BA -0.874071 -0.485000  0.027834 -0.485130  0.874439  0.002351 -0.025480 -0.011448 -0.999610    45.305   -12.563    15.363
 reverse transforms 
TRANS_AB -0.874071 -0.485130 -0.025480 -0.485000  0.874439 -0.011448  0.027834  0.002351 -0.999610    33.896    33.134    14.126
 
 Setting up LSSR interatomic distance restraints
 Interpreting card: NOTE BUSTER_SIM_RESTRAIN_LSSR ncsautoAB 6.666
        setting up interatomic distance pair restraints with:
        Dmin=         0.00
        Dmax=         5.50
        sigma=        0.20
        Vmax=         3.00
        weight=       6.67
        number of distance pairs defined for this set is      34023
 Have not supplied either '-sim_swap_equiv' or '-sim_swap_equiv_plus' argument 
   so will not try swaping equivalent atoms to improve NCS and/or target agreement.
 
 NCS by LSSR outlier analysis
 -autoncs option automatic NCS prune routine -------- 
 Residues pruned out by gradient condition:
          A|148     A|149     A|150     A|151     A|173     A|182     A|186
          A|187     A|188     A|189     A|201     A|215     A|216     A|217
          A|219     A|220     A|223     A|226     A|230     A|256     A|259
          A|264     A|303     A|307     A|344     A|345     B|148     B|149
          B|150     B|151     B|173     B|182     B|186     B|187     B|188
          B|189     B|201     B|215     B|216     B|217     B|219     B|220
          B|223     B|226     B|230     B|256     B|259     B|264     B|303
          B|307     B|344     B|345
  
 Residues pruned out by function/max condition:
          A|149!    A|187!    A|188!    A|215!    A|264!    A|345!   A|ENDA 
          B|149!    B|187!    B|188!    B|215!    B|264!    B|345!   B|ENDA 
       
 Total number of residues for which LSSR restraints have been pruned out is       54 out of      698 participating
 Number of LSSR restraints pruned out is      3593 this is   10.6% of the total           34023
  
 -autoncs .Gelly cards suitable for adaptation for manual definition/pruning
           (note auto pruning is done in a slightly different way so exact results may differ slightly)
 -autoncs option .Gelly cards also written to file  auto_gelly_cards.txt
NOTE BUSTER_SET ncsautoXcld = Water    # card added - produced by -autoncs
NOTE BUSTER_SET ncsautoXcld = ncsautoXcld + { B|148 B|149 B|150 B|151 B|173 }    # card added - produced by -autoncs
NOTE BUSTER_SET ncsautoXcld = ncsautoXcld + { B|182 B|186 B|187 B|188 B|189 }    # card added - produced by -autoncs
NOTE BUSTER_SET ncsautoXcld = ncsautoXcld + { B|201 B|215 B|216 B|217 B|219 }    # card added - produced by -autoncs
NOTE BUSTER_SET ncsautoXcld = ncsautoXcld + { B|220 B|223 B|226 B|230 B|256 }    # card added - produced by -autoncs
NOTE BUSTER_SET ncsautoXcld = ncsautoXcld + { B|259 B|264 B|303 B|307 B|344 }    # card added - produced by -autoncs
NOTE BUSTER_SET ncsautoXcld = ncsautoXcld + { B|345 B|ENDA }    # card added - produced by -autoncs
NOTE BUSTER_SET ncsautoABset = Chain_B \ ncsautoXcld    # card added - produced by -autoncs
NOTE BUSTER_SIM_DEFINE ncsautoAB ncsautoABset A    # card added - produced by -autoncs
NOTE BUSTER_SIM_RESTRAIN_LSSR ncsautoAB 6.666    # card added - produced by -autoncs

 LSSR function contribution before pruning     28377.3
                             after pruning     10871.2
 Graph of which residues pruned from LSSR: ncs_pruned.mtv
 Graph of LSSR function outliers ncs_function_outliers.mtv
 Graph of LSSR gradient outliers ncs_gradient_outliers.mtv
 LSSR - analysis of distance difference distribution for NCS
         DeltaD less than 0.5 Angs:  proportion of population= 98.59% average DeltaD of subset     0.010 Angs
 Histogram of LSSR deltaD distribution ncs_histogram.mtv
 
 Interpreting card: NOTE BUSTER_TLS_SET tls1 { A|* }
 Interpreting card: NOTE BUSTER_TLS_SET tls2 { B|* }
 Interpreting card: NOTE BUSTER_TLS_SET tls1 { A|* }
 Interpreting card: NOTE BUSTER_TLS_T   tls1     -6.89402E-02  9.73396E-02  1.12143E-02 -5.14314E-02  3.70007E-02 -6.09601E-02
 Interpreting card: NOTE BUSTER_TLS_L   tls1      2.34061E+00  1.26720E+00  1.13030E+00 -7.76197E-01 -1.07598E+00  5.55382E-01
 Interpreting card: NOTE BUSTER_TLS_S   tls1      2.71047E-02 -3.50758E-01  5.52083E-01 -3.65020E-01  2.02419E-01 -2.02943E-01  6.79091E-03 -2.19839E-01
 Interpreting card: NOTE BUSTER_TLS_O   tls1     -2.32263E+01 -8.41675E+01  1.09752E+01
 Interpreting card: NOTE BUSTER_TLS_SET tls2 { B|* }
 Interpreting card: NOTE BUSTER_TLS_T   tls2     -1.17934E-01 -4.34381E-02  3.08544E-02  1.57672E-02  3.71279E-02 -2.24165E-02
 Interpreting card: NOTE BUSTER_TLS_L   tls2      1.51304E+00  9.47989E-01  1.07060E+00  6.77124E-01 -9.34394E-01 -7.04506E-01
 Interpreting card: NOTE BUSTER_TLS_S   tls2     -4.36251E-03  8.11357E-02 -1.53271E-01  1.51330E-01  1.63309E-01  5.90040E-02 -7.47994E-02  3.57312E-02
 Interpreting card: NOTE BUSTER_TLS_O   tls2      2.22079E+01 -7.23190E+01  3.56420E+00
 Interpreting card: NOTE BUSTER_TLS_FIX tls1 ALL
 Interpreting card: NOTE BUSTER_TLS_FIX tls2 ALL


 TLS information. Total number of groups:     2


 Group   Natoms    FixRB   FixTLS   Group Tag    Group Spec
     1     2768      Y        Y     tls1    { A|* }
     2     2770      Y        Y     tls2    { B|* }
 
NOTE BUSTER_TLS_SET tls1 { A|* }
NOTE BUSTER_TLS_T   tls1     -6.89402E-02  9.73396E-02  1.12143E-02 -5.14314E-02  3.70007E-02 -6.09601E-02
NOTE BUSTER_TLS_L   tls1      2.34061E+00  1.26720E+00  1.13030E+00 -7.76197E-01 -1.07598E+00  5.55382E-01
NOTE BUSTER_TLS_S   tls1      2.71047E-02 -3.50758E-01  5.52083E-01 -3.65020E-01  2.02419E-01 -2.02943E-01  6.79091E-03 -2.19839E-01
NOTE BUSTER_TLS_O   tls1     -2.32263E+01 -8.41675E+01  1.09752E+01
NOTE BUSTER_TLS_FIX tls1 ALL
 
NOTE BUSTER_TLS_SET tls2 { B|* }
NOTE BUSTER_TLS_T   tls2     -1.17934E-01 -4.34381E-02  3.08544E-02  1.57672E-02  3.71279E-02 -2.24165E-02
NOTE BUSTER_TLS_L   tls2      1.51304E+00  9.47989E-01  1.07060E+00  6.77124E-01 -9.34394E-01 -7.04506E-01
NOTE BUSTER_TLS_S   tls2     -4.36251E-03  8.11357E-02 -1.53271E-01  1.51330E-01  1.63309E-01  5.90040E-02 -7.47994E-02  3.57312E-02
NOTE BUSTER_TLS_O   tls2      2.22079E+01 -7.23190E+01  3.56420E+00
NOTE BUSTER_TLS_FIX tls2 ALL


 TLS: mmCIF group specification begin


#
loop_
_pdbx_refine_tls_group.id
_pdbx_refine_tls_group.refine_tls_id
_pdbx_refine_tls_group.beg_PDB_ins_code
_pdbx_refine_tls_group.beg_auth_asym_id
_pdbx_refine_tls_group.beg_auth_seq_id
_pdbx_refine_tls_group.end_PDB_ins_code
_pdbx_refine_tls_group.end_auth_asym_id
_pdbx_refine_tls_group.end_auth_seq_id
_pdbx_refine_tls_group.selection
_pdbx_refine_tls_group.selection_details
   1   1 . A   -1 . A  346 . '{ A|* }'
   2   1 . A  501 . A  501 . '{ A|* }'
   3   2 . B   -1 . B  346 . '{ B|* }'
   4   2 . B  401 . B  401 . '{ B|* }'
#


 TLS: mmCIF group specification end


 Interpreting card: NOTE BUSTER_ANISO_SET Empty
 Interpreting card: NOTE BUSTER_ANISO_WEIGHT SCA  16.0
 Interpreting card: NOTE BUSTER_ANISO_WEIGHT SPH   0.6    0.6
 Interpreting card: NOTE BUSTER_ANISO_WEIGHT SIM   7.8    4.8    2.6    0
 Interpreting card: NOTE BUSTER_ANISO_WEIGHT RIG  21.5   21.5    3.7    0


 After TLS and ANISO: Number of atoms with fixed/combined xyz =     0
 After TLS and ANISO: Number of atoms with fixed/combined B   =     0
 After TLS and ANISO: Number of atoms with fixed/combined OCC =  6119
 After TLS and ANISO: Number of atoms with fixed/combined BIJ =  6119
 After TLS and ANISO: Number of atoms in a TLS group          =  5538
                                     cf total number of atoms =  6119
 

 ############### Setup state vector

 No free_vect_ulim_xyz argument found: using default of  100.000
 No free_vect_llim_xyz argument found: using default of -100.000
 Number of parameters added for TLS description                 =       0
 Number of parameters to describe molecular conformation        =   24476

 ############### Setup free vector

 Number of free parameters to be adjusted in refinement   =   24476

 ############### Setting up own geometry


 No command line argument '-tnt_fn' so will use gelly to do geometry function calculations

 ============= Call TNT functions


 ============= Get TNT symmetry information

 
      Getting symmetry operators from TNT. 
          gelly will classify symmetry using pdb-like convention: 
               SYMOP   SYMMETRY
              NNNMMM   OPERATOR
                1555   X,Y,Z
                2555   -Y,X-Y,Z
                3555   -X+Y,-X,Z
                4555   -X,-Y,1/2+Z
                5555   Y,-X+Y,1/2+Z
                6555   X-Y,X,1/2+Z
          where NNN -> operator number and MMM -> translation vector
 

 ============= Own Gelly geometry/restraints setup


 ----------- Identify bond-types


    Setting up GEOMBONDREC_TYPE_HASH* to be able to map a given bond type (e.g.
    "single") to an internal integer (GBOND_TYPE=1) etc.

    Using default  GEOMBONDREC_TYPE_HASH_STR='SING* 1 DOUB* 2 TRIP* 3 AROM* 4 DELO* 4 METAL* 5':
    to override default use a NOTE BUSTER_GEOMBONDREC_TYPE_HASH_STR gelly card.

 ----------- Classify torsion into peptide omega and other


 ----------- Setup of non-bonded terms

 Ideal-distance contact term setup:
    Restraint function constants (normally these values are from NOTE BUSTER_IDEAL_CONTACT_* cards)
    Sigma for general contacts      0.200 Angs
    Sigma for 1-4 contacts          0.100 Angs (contacts across a torsion)
    Sigma for 1-5 contacts          0.150 Angs
    Sigma for 1-N contacts          0.200 Angs
    Sigma for HBOND pairs           0.100 Angs (contacts between HB_types A and D or B's)
    Contact distance increment for each atom in HBOND          -0.150 Angs 
    Contact distance increment for H-bond acceptor              0.100 Angs 
    Contact distance increment for 1-4 with an O atom          -0.050 Angs 
    Contact distance increment for 1-4 with an N atom          -0.180 Angs 
    Contact distance increment for 1-4 with an C atom          -0.370 Angs 
    Contact distance increment for 1-4 with an S atom          -0.150 Angs 
    Contact distance increment for 1-4 with an P atom          -0.150 Angs 
    Contact distance increment for 1-4 with any other atom     -0.150 Angs 
    All atoms matched for CCP4 atom type.
    Number of CCP4 atom types used is         24 (H-bound and not H-bound counted separately)

 Setting up short/ideal-distance contacts
   Using gelly special position handling. (For TNT instead rerun with argument '-special_tnt')

   No command line argument '-special_all' specified. We will auto-generate
   EXCLUDE cards only for atom-atom contacts across symmetry (i.e. on special
   position) when this involves a contact within a residue to itself.

   Have set up non-bonded exclusions - 1-3 contacts
   Atom A|27:CA         makes the most 1-3 contacts:  6

   Have set up non-bonded exclusions - 1-4 contacts
   Atom A|27:N          makes the most 1-4 contacts:  7

   Have set up non-bonded exclusions - 1-5 contacts
   Atom A|28:C.A        makes the most 1-5 contacts: 10

 ############################################################################################################
 ###################################################### setting up contact terms
 ############################################################################################################


 ============= Call Gelly versions of TNT functions

 WARNING: 1-4 contact without 1-(2,3)-4 data for A|501:C01(LIG)       and A|501:O04(LIG)       X-X
 WARNING: 1-4 contact without 1-(2,3)-4 data for A|501:N06(LIG)       and A|501:N09(LIG)       X-X
 WARNING: 1-4 contact without 1-(2,3)-4 data for A|501:C07(LIG)       and A|501:C10(LIG)       X-X
 WARNING: 1-4 contact without 1-(2,3)-4 data for A|501:C08(LIG)       and A|501:C11(LIG)       X-X
 WARNING: 1-4 contact without 1-(2,3)-4 data for B|401:C1(CIT)        and B|401:C4(CIT)        X-X
 WARNING: 1-4 contact without 1-(2,3)-4 data for B|401:C2(CIT)        and B|401:O6(CIT)        X-X
 WARNING: 1-4 contact without 1-(2,3)-4 data for B|401:O7(CIT)        and B|401:O5(CIT)        X-X
 WARNING: 1-4 contact without 1-(2,3)-4 data for B|401:C4(CIT)        and B|401:O6(CIT)        X-X

       TERM      weight      number      TNT_fn    Gelly_fn        diff
       BOND       2.000        5653     791.167     791.167       0.000
       ANGL       2.000        7670    4012.276    4012.276       0.000
       IMPR       0.000           0       0.000       0.000       0.000
       TORS       2.000        1968    2849.405    2849.405      -0.000
       TRIG       2.000           0       0.000       0.000       0.000
       PLAN       5.000         963    1900.824    1900.824       0.000
       CONT       5.000           0      36.212       0.000      36.212
       BCOR      10.000        5630    3261.923    6932.475   -3670.553


 ============= Call Gelly geometry functions

   Switching torsion functional form to sinusoidal rather than split harmonic functional.
      if you want this not to happen then rerun with '-torsharm'
      Weight for sinusoidal torsion=     2.000
   BCOR function/gradient will be split between H and non-H atoms (as for any atom pair). Use '-bcorrel_all_in_hydrogens' to change this.

 ----------- Fix B-correlation restraints

 
 Checking for bonds that do not have a bcorrel restraint defined
 We will wipe all existing BCORrels if OCC=0.0 atom(s) is/are involved. This can be' switched off via the '-bcorrel_nowipe_zeroocc' flag.
 We will wipe all existing BCORrels if only non-hydrogen atom(s) with fixed B(iso) are involved. This can be switched off via the '-bcorrel_nowipe_fixb_nonhydrogens' flag.
 We will keep all existing BCORrels if hydrogen atom(s) with fixed B(iso) is/are involved. This can be switched off via the '-bcorrel_wipe_fixb_hydrogens' flag.
 No -bcorrel_missing_sigma found. So will set sigma to     2.5 Ang**2 for any bond with no existing BCORrel
 No -bcorrel_missing_sigma_hydrogens found. So will set sigma to     1.0 Ang**2 for any bond with no existing BCORrel involving hydrogens

 No active BCORels found involving OCC=0.0 atom(s)


 No active BCORels found involving fixed-B (only) non-hydrogen atom(s)

    add a default BCOR for bond A|501:C01(LIG) A|501:N02(LIG)
    add a default BCOR for bond A|501:N02(LIG) A|501:C03(LIG)
    add a default BCOR for bond A|501:C03(LIG) A|501:O04(LIG)
    add a default BCOR for bond A|501:C03(LIG) A|501:C05(LIG)
    add a default BCOR for bond A|501:C05(LIG) A|501:N06(LIG)
    add a default BCOR for bond A|501:N06(LIG) A|501:C07(LIG)
    add a default BCOR for bond A|501:N06(LIG) A|501:C11(LIG)
    add a default BCOR for bond A|501:C07(LIG) A|501:C08(LIG)
    add a default BCOR for bond A|501:C08(LIG) A|501:N09(LIG)
    add a default BCOR for bond A|501:N09(LIG) A|501:C10(LIG)
    add a default BCOR for bond A|501:C10(LIG) A|501:C11(LIG)
    add a default BCOR for bond B|401:C1(CIT) B|401:C2(CIT)
    add a default BCOR for bond B|401:C1(CIT) B|401:O1(CIT)
    add a default BCOR for bond B|401:C1(CIT) B|401:O2(CIT)
    add a default BCOR for bond B|401:C2(CIT) B|401:C3(CIT)
    add a default BCOR for bond B|401:C3(CIT) B|401:C4(CIT)
    add a default BCOR for bond B|401:C3(CIT) B|401:C6(CIT)
    add a default BCOR for bond B|401:C3(CIT) B|401:O7(CIT)
    add a default BCOR for bond B|401:C4(CIT) B|401:C5(CIT)
    add a default BCOR for bond B|401:C5(CIT) B|401:O3(CIT)
    add a default BCOR for bond B|401:C5(CIT) B|401:O4(CIT)
    add a default BCOR for bond B|401:C6(CIT) B|401:O5(CIT)
    add a default BCOR for bond B|401:C6(CIT) B|401:O6(CIT)

 Have added a total of        23 BCORrels for bonds which do not already have a BCORrel


 No bonds (for potential BCORrel addition) were skipped due to both atoms having constant/fixed B(iso)


 ----------- Deactivate hydrogen-free planes if hydrogen atoms present

 A total of       0 planes have been deactivated because they are duplicated hydrogen-free and with-hydrogen forms.

 ----------- Handle peptide omega angle(s)

      Torsion angles split into peptide omega and other
         Number of peptide omega angles is       698
         Number of other torsion is             1270
      Peptide omega angles are monitored rather than directly restrained (PLANE used instead).

 ----------- Fix cis-PRO

 Now apply the fix to get correct restraints for cis PRO residues
   Have got      11 bonds records and      26 angles
   from GEOMETRY cards for the special PROC residue
   Weight for ideal-distance contact term=     4.000

 ----------- Prepare SSBOND/LINK for output PDB file


 ----------- Prepare for QM


 ----------- Prepare symmetry in utility distance restraints


 ############### Setting up Rhofit contacts (if needed)

 No ligand-(fixed protein) contact term will be used

 ############### Working out initial function and gradient


   MaxLik Scaling Cycle 0002
 
 Initial functional value = 4.144846E+05 rms gradient=      86.2538
 X-ray contribution to function value = 3.792878E+05
 Geometry contribution (inc ncs)      =      35196.7
 Geometry function summary
     term                           number     weight       rms        GooF      function     fn/numb
     ================================================================================================
     BOND bond lengths (angs)       5653       2.000      0.0053       0.265       791.17       0.140
     ANGL bond angles (degs)        7670       2.000        0.86       0.511      4012.28       0.523
     TORS split-harmonic tors       1270       0.000       16.84       1.059         0.00       0.000
     SINTOR sinusoidal tors         1270       2.000       16.84                  2138.29       1.684
     PLAN general plane (angs)       963       5.000      0.0126       0.628      1900.82       1.974
     BCORrelations (angs^2)         5653      10.000       0.982       0.351      6948.50       1.229
     CONT (bad contacts)               0       5.000                                 0.00
     IDEAL(ideal-dist contact)      5142       4.000                              8500.17
     CHIRAL (gelly semiharmon)       740       5.000                                34.37
     SIM similarity restraint for NCS and/or TARGET                              10871.15
     OCCSUM restraint on sum of occupanies                                           0.00       as none defined
     DISTAN utility restraints interatomic distance                                  0.00       as none defined
     UTILANGLE utility angle restraints                                              0.00       as none defined
     UTILTOR utility restraints on torsion angles                                    0.00       as none defined
     Peptide omega torsion angles                           2.68 degs - monitor only

 Weighted rms      0.0053         0.858  (temporary rms bond and angle for BUSTER to pick up!)
 Weighted intra-residual rms(bond) for StdProtein (excluding H)      0.0051
 Weighted intra-residual rms(bond) for Buffer     (excluding H)      0.0027

 ############### Running initial check


 Initial Geometry Sanity Check

 Check for duplicate bonds: no duplicates found
 
 Check for duplicate or missing bond angles: all are OK
 Check for bond between an atom and itself not involving symmetry:  all are OK
   There are no bad contacts where Delta/sigma is greater than     6.5
   Worst contact dist=   2.777 std=   3.603 sigma=   0.198 abs(Delta/sigma)=    4.174 ats A|104:CE (MET) S|459:O (HOH)
   It is likely that the geometry libraries, sequence info and links match the input molecule
 GEOMETRY CHECK OVERALL:  PASS


 ############### Creating geometry/screen report


 ############### Option "tao" - run Tao optimizer

 TAO: Toolkit for Advanced Optimization. Reference: 
     Munson, T., Sarich, J., Wild, S., Benson, S., McInnes, L.C. (2018)
     "TAO User Manual (Revision 3.9)", Mathematics and Computer Science
     Division, Argonne National Laboratory
     ANL/MCS-TM-322, http://www.mcs.anl.gov/petsc/
 
 Have found -keeppdb flag so will write out a .pdb file every 1000000 its during opt
 Will apply TAO optimization 
 Maximum number of iterations      100
 Convergence test on Grms       4.0000 (-glim)
 Limit on rms displacement to original position (rmsD2init): NONE test inactive (-dlim)

    analysing argument no 0 : -autoncs with j=0
    analysing argument no 1 : -hydrogens_input_occ2zero with j=0
    analysing argument no 2 : -hydrogens_output_occ2heavy with j=0
    analysing argument no 3 : -screen with j=0
    analysing argument no 4 : 100 with j=0
    analysing argument no 5 : -screen_sigma with j=0
    analysing argument no 6 : 3.0 with j=0
    analysing argument no 7 : -keeppdb with j=0
    analysing argument no 8 : 1000000 with j=0
    analysing argument no 9 : -taomonitor_verbose with j=0
    found argument starting with "-tao" : -taomonitor_verbose
    analysing argument no 10 : 2 with j=1
    found value after -tao/-options_/-viewer_ argument : "2"
 PETSC/TAO: setting option "-taomonitor_verbose" = "2"


   MaxLik Scaling Cycle 0003
   Iters     Tot_Funct    Xray_Funct    Geom_Funct          Grms     rmsD2init     maxD2init             R         Rfree           LLG       LLGfree     rms(bond)    rms(angle)       Opttag_H
   Iters             K   B_IMPF_FRAG        K_MISS   B_TEMP_MISS   K_IMPF_MISS   B_IMPF_MISS        K_SOLV   B_TEMP_SOLV   K_IMPF_SOLV   B_IMPF_SOLV        B_TEMP     B_TEMP_11     B_TEMP_12     B_TEMP_22     B_TEMP_13     B_TEMP_23     B_TEMP_33      K_WILSON      B_WILSON      FGCYCLES       Scatag_H
   Iters          BOND         ANGLE      IMPROPER       TORSION    SINTORSION      TRIGONAL        PLANAR  BCORRELATION       CONTACT     IDEALDIST        CHIRAL    SIMILARITY       OCCSSUM  UTILDISTANCE     UTILANGLE   UTILTORSION        RHOFIT        ADP-01        ADP-02        ADP-03        ADP-04        ADP-05        ADP-06        ADP-07        ADP-08        ADP-09        ADP-10      RHOFITPC            QM       Funtag_H
       0  4.142917E+05  3.792880E+05       35003.7       81.1307  0.000000E+00  0.000000E+00      0.213621      0.233912  0.000000E+00  0.000000E+00    0.00530817      0.858054       Opttag_D
       0       71.5915       2.32060       1.00000  0.000000E+00       1.00000  0.000000E+00      0.846011       39.9615       1.00000       42.8686  0.000000E+00       3.97931  0.000000E+00       3.97931  0.000000E+00  0.000000E+00      -7.95862       72.3087       2.35054        2       Scatag_D
       0       791.167       4012.28  0.000000E+00  0.000000E+00       2138.29  0.000000E+00       1900.82       6755.42  0.000000E+00       8500.17       34.3716       10871.2 -1.000000E+10 -1.000000E+10 -1.000000E+10 -1.000000E+10 -1.000000E+10  0.000000E+00  0.000000E+00  0.000000E+00  0.000000E+00  0.000000E+00  0.000000E+00  0.000000E+00  0.000000E+00  0.000000E+00  0.000000E+00  0.000000E+00  0.000000E+00       Funtag_D
   Geometry Restraint Deviations SCREEN Information

 

   MaxLik Scaling Cycle 0004

   MaxLik Scaling Cycle 0005

   MaxLik Scaling Cycle 0006

   Wilson plot

   Statistics in resolution bins after refinement cycle 0001
```


|  |  |
| --- | --- |
|  | - Rxpct factors plot- Rxpct factors table- <Log-Lik Gain>- Ln Sigma A- Correlation Coefficients |


```
   Iters     Tot_Funct    Xray_Funct    Geom_Funct          Grms     rmsD2init     maxD2init             R         Rfree           LLG       LLGfree     rms(bond)    rms(angle)
   Iters             K   B_IMPF_FRAG        K_MISS   B_TEMP_MISS   K_IMPF_MISS   B_IMPF_MISS        K_SOLV   B_TEMP_SOLV   K_IMPF_SOLV   B_IMPF_SOLV        B_TEMP     B_TEMP_11     B_TEMP_12     B_TEMP_22     B_TEMP_13     B_TEMP_23     B_TEMP_33      K_WILSON      B_WILSON      FGCYCLES
   Iters          BOND         ANGLE      IMPROPER       TORSION    SINTORSION      TRIGONAL        PLANAR  BCORRELATION       CONTACT     IDEALDIST        CHIRAL    SIMILARITY       OCCSSUM  UTILDISTANCE     UTILANGLE   UTILTORSION        RHOFIT        ADP-01        ADP-02        ADP-03        ADP-04        ADP-05        ADP-06        ADP-07        ADP-08        ADP-09        ADP-10      RHOFITPC            QM
       1  4.129824E+05  3.785191E+05       34463.3       64.8876  7.257778E-04    0.00431622      0.213364      0.233980    0.00174090  1.086997E-04    0.00546430      0.858856       Opttag_D
       1       71.5803       2.28707       1.00000  0.000000E+00       1.00000  0.000000E+00      0.845400       39.8664       1.00000       43.0301  0.000000E+00       3.97576  0.000000E+00       3.97576  0.000000E+00  0.000000E+00      -7.95153       72.3087       2.35054        3       Scatag_D
       1       838.390       4019.77  0.000000E+00  0.000000E+00       2137.47  0.000000E+00       1916.07       6122.84  0.000000E+00       8507.95       34.4233       10886.4 -1.000000E+10 -1.000000E+10 -1.000000E+10 -1.000000E+10 -1.000000E+10  0.000000E+00  0.000000E+00  0.000000E+00  0.000000E+00  0.000000E+00  0.000000E+00  0.000000E+00  0.000000E+00  0.000000E+00  0.000000E+00  0.000000E+00  0.000000E+00       Funtag_D
   Geometry Restraint Deviations SCREEN Information

 

   MaxLik Scaling Cycle 0007

   Wilson plot

   Statistics in resolution bins after refinement cycle 0002
```


|  |  |
| --- | --- |
|  | - Rxpct factors plot- Rxpct factors table- <Log-Lik Gain>- Ln Sigma A- Correlation Coefficients |


```
   Iters     Tot_Funct    Xray_Funct    Geom_Funct          Grms     rmsD2init     maxD2init             R         Rfree           LLG       LLGfree     rms(bond)    rms(angle)
   Iters             K   B_IMPF_FRAG        K_MISS   B_TEMP_MISS   K_IMPF_MISS   B_IMPF_MISS        K_SOLV   B_TEMP_SOLV   K_IMPF_SOLV   B_IMPF_SOLV        B_TEMP     B_TEMP_11     B_TEMP_12     B_TEMP_22     B_TEMP_13     B_TEMP_23     B_TEMP_33      K_WILSON      B_WILSON      FGCYCLES
   Iters          BOND         ANGLE      IMPROPER       TORSION    SINTORSION      TRIGONAL        PLANAR  BCORRELATION       CONTACT     IDEALDIST        CHIRAL    SIMILARITY       OCCSSUM  UTILDISTANCE     UTILANGLE   UTILTORSION        RHOFIT        ADP-01        ADP-02        ADP-03        ADP-04        ADP-05        ADP-06        ADP-07        ADP-08        ADP-09        ADP-10      RHOFITPC            QM
       2  4.122065E+05  3.777866E+05       34420.0       61.7410    0.00168369     0.0107423      0.213015      0.233941    0.00346234  4.108693E-04    0.00570778      0.862433       Opttag_D
       2       71.5637       2.25677       1.00000  0.000000E+00       1.00000  0.000000E+00      0.845011       39.8048       1.00000       43.1236  0.000000E+00       3.97284  0.000000E+00       3.97284  0.000000E+00  0.000000E+00      -7.94567       72.3087       2.35054        1       Scatag_D
       2       914.771       4053.33  0.000000E+00  0.000000E+00       2136.24  0.000000E+00       1941.48       5899.01  0.000000E+00       8522.74       34.5813       10917.8 -1.000000E+10 -1.000000E+10 -1.000000E+10 -1.000000E+10 -1.000000E+10  0.000000E+00  0.000000E+00  0.000000E+00  0.000000E+00  0.000000E+00  0.000000E+00  0.000000E+00  0.000000E+00  0.000000E+00  0.000000E+00  0.000000E+00  0.000000E+00       Funtag_D
   Geometry Restraint Deviations SCREEN Information

 
       3  4.116702E+05  3.770303E+05       34639.8       58.2812    0.00273751     0.0181088      0.212655      0.233885    0.00523939  7.643678E-04    0.00596502      0.866135       Opttag_D
       3       71.5477       2.22650       1.00000  0.000000E+00       1.00000  0.000000E+00      0.844637       39.7467       1.00000       43.2170  0.000000E+00       3.96916  0.000000E+00       3.96916  0.000000E+00  0.000000E+00      -7.93832       72.3087       2.35054        1       Scatag_D
       3       999.084       4088.20  0.000000E+00  0.000000E+00       2134.90  0.000000E+00       1973.54       5905.50  0.000000E+00       8541.78       34.6375       10962.2 -1.000000E+10 -1.000000E+10 -1.000000E+10 -1.000000E+10 -1.000000E+10  0.000000E+00  0.000000E+00  0.000000E+00  0.000000E+00  0.000000E+00  0.000000E+00  0.000000E+00  0.000000E+00  0.000000E+00  0.000000E+00  0.000000E+00  0.000000E+00       Funtag_D
       4  4.112019E+05  3.761271E+05       35074.8       49.6813    0.00407472     0.0276997      0.212216      0.233791    0.00736179    0.00126276    0.00625236      0.867407       Opttag_D
       4       71.5316       2.19164       1.00000  0.000000E+00       1.00000  0.000000E+00      0.844218       39.6874       1.00000       43.3212  0.000000E+00       3.96447  0.000000E+00       3.96447  0.000000E+00  0.000000E+00      -7.92893       72.3087       2.35054        1       Scatag_D
       4       1097.65       4100.22  0.000000E+00  0.000000E+00       2133.12  0.000000E+00       2018.87       6092.25  0.000000E+00       8571.00       34.6727       11027.0 -1.000000E+10 -1.000000E+10 -1.000000E+10 -1.000000E+10 -1.000000E+10  0.000000E+00  0.000000E+00  0.000000E+00  0.000000E+00  0.000000E+00  0.000000E+00  0.000000E+00  0.000000E+00  0.000000E+00  0.000000E+00  0.000000E+00  0.000000E+00       Funtag_D
       5  4.107850E+05  3.754055E+05       35379.5       42.4005    0.00530887     0.0366575      0.211851      0.233688    0.00905746    0.00176295    0.00644495      0.870246       Opttag_D
       5       71.5220       2.16527       1.00000  0.000000E+00       1.00000  0.000000E+00      0.843941       39.6556       1.00000       43.3899  0.000000E+00       3.96066  0.000000E+00       3.96066  0.000000E+00  0.000000E+00      -7.92133       72.3087       2.35054        1       Scatag_D
       5       1166.32       4127.10  0.000000E+00  0.000000E+00       2131.35  0.000000E+00       2064.13       6167.55  0.000000E+00       8600.64       34.7135       11087.7 -1.000000E+10 -1.000000E+10 -1.000000E+10 -1.000000E+10 -1.000000E+10  0.000000E+00  0.000000E+00  0.000000E+00  0.000000E+00  0.000000E+00  0.000000E+00  0.000000E+00  0.000000E+00  0.000000E+00  0.000000E+00  0.000000E+00  0.000000E+00       Funtag_D
       6  4.103086E+05  3.743432E+05       35965.4       51.7748    0.00744344     0.0518840      0.211286      0.233485     0.0115538    0.00263177    0.00669223      0.880114       Opttag_D
       6       71.5160       2.12913       1.00000  0.000000E+00       1.00000  0.000000E+00      0.843624       39.6346       1.00000       43.4708  0.000000E+00       3.95489  0.000000E+00       3.95489  0.000000E+00  0.000000E+00      -7.90978       72.3087       2.35054        1       Scatag_D
       6       1257.53       4221.23  0.000000E+00  0.000000E+00       2128.52  0.000000E+00       2145.62       6332.10  0.000000E+00       8657.18       34.9914       11188.3 -1.000000E+10 -1.000000E+10 -1.000000E+10 -1.000000E+10 -1.000000E+10  0.000000E+00  0.000000E+00  0.000000E+00  0.000000E+00  0.000000E+00  0.000000E+00  0.000000E+00  0.000000E+00  0.000000E+00  0.000000E+00  0.000000E+00  0.000000E+00       Funtag_D
       7  4.099505E+05  3.735464E+05       36404.1       57.1801    0.00939190     0.0653400      0.210839      0.233276     0.0134262    0.00344196    0.00683352      0.886633       Opttag_D
       7       71.5216       2.10440       1.00000  0.000000E+00       1.00000  0.000000E+00      0.843453       39.6411       1.00000       43.5226  0.000000E+00       3.95022  0.000000E+00       3.95022  0.000000E+00  0.000000E+00      -7.90045       72.3087       2.35054        1       Scatag_D
       7       1311.19       4284.00  0.000000E+00  0.000000E+00       2125.80  0.000000E+00       2220.48       6450.02  0.000000E+00       8712.12       35.5664       11264.9 -1.000000E+10 -1.000000E+10 -1.000000E+10 -1.000000E+10 -1.000000E+10  0.000000E+00  0.000000E+00  0.000000E+00  0.000000E+00  0.000000E+00  0.000000E+00  0.000000E+00  0.000000E+00  0.000000E+00  0.000000E+00  0.000000E+00  0.000000E+00       Funtag_D
       8  4.096448E+05  3.733236E+05       36321.2       42.0036     0.0101377     0.0701729      0.210703      0.233172     0.0139496    0.00379857    0.00680808      0.884211       Opttag_D
       8       71.5298       2.09863       1.00000  0.000000E+00       1.00000  0.000000E+00      0.843435       39.6545       1.00000       43.5366  0.000000E+00       3.94922  0.000000E+00       3.94922  0.000000E+00  0.000000E+00      -7.89845       72.3087       2.35054        1       Scatag_D
       8       1301.45       4260.62  0.000000E+00  0.000000E+00       2124.72  0.000000E+00       2246.12       6343.96  0.000000E+00       8734.61       35.9687       11273.7 -1.000000E+10 -1.000000E+10 -1.000000E+10 -1.000000E+10 -1.000000E+10  0.000000E+00  0.000000E+00  0.000000E+00  0.000000E+00  0.000000E+00  0.000000E+00  0.000000E+00  0.000000E+00  0.000000E+00  0.000000E+00  0.000000E+00  0.000000E+00       Funtag_D
       9  4.092654E+05  3.728459E+05       36419.5       35.9568     0.0117600     0.0801070      0.210420      0.232964     0.0150720    0.00450383    0.00680326      0.886817       Opttag_D
       9       71.5514       2.08641       1.00000  0.000000E+00       1.00000  0.000000E+00      0.843360       39.6835       1.00000       43.5783  0.000000E+00       3.94651  0.000000E+00       3.94651  0.000000E+00  0.000000E+00      -7.89302       72.3087       2.35054        1       Scatag_D
       9       1299.61       4285.77  0.000000E+00  0.000000E+00       2122.62  0.000000E+00       2298.65       6299.19  0.000000E+00       8784.20       36.9216       11292.5 -1.000000E+10 -1.000000E+10 -1.000000E+10 -1.000000E+10 -1.000000E+10  0.000000E+00  0.000000E+00  0.000000E+00  0.000000E+00  0.000000E+00  0.000000E+00  0.000000E+00  0.000000E+00  0.000000E+00  0.000000E+00  0.000000E+00  0.000000E+00       Funtag_D
      10  4.089860E+05  3.721855E+05       36800.5       48.9507     0.0138991     0.0924579      0.210050      0.232704     0.0166239    0.00535057    0.00688615      0.897321       Opttag_D
      10       71.5814       2.06891       1.00000  0.000000E+00       1.00000  0.000000E+00      0.843181       39.7149       1.00000       43.6492  0.000000E+00       3.94154  0.000000E+00       3.94154  0.000000E+00  0.000000E+00      -7.88307       72.3087       2.35054        1       Scatag_D
      10       1331.47       4387.90  0.000000E+00  0.000000E+00       2119.92  0.000000E+00       2365.06       6378.43  0.000000E+00       8851.58       37.9143       11328.2 -1.000000E+10 -1.000000E+10 -1.000000E+10 -1.000000E+10 -1.000000E+10  0.000000E+00  0.000000E+00  0.000000E+00  0.000000E+00  0.000000E+00  0.000000E+00  0.000000E+00  0.000000E+00  0.000000E+00  0.000000E+00  0.000000E+00  0.000000E+00       Funtag_D
      11  4.086836E+05  3.715038E+05       37179.8       38.2521     0.0162831      0.108155      0.209660      0.232450     0.0182260    0.00618607    0.00695957      0.898781       Opttag_D
      11       71.6179       2.05169       1.00000  0.000000E+00       1.00000  0.000000E+00      0.842999       39.7582       1.00000       43.7221  0.000000E+00       3.93602  0.000000E+00       3.93602  0.000000E+00  0.000000E+00      -7.87204       72.3087       2.35054        1       Scatag_D
      11       1360.01       4402.20  0.000000E+00  0.000000E+00       2117.18  0.000000E+00       2435.97       6527.50  0.000000E+00       8928.63       38.8701       11369.4 -1.000000E+10 -1.000000E+10 -1.000000E+10 -1.000000E+10 -1.000000E+10  0.000000E+00  0.000000E+00  0.000000E+00  0.000000E+00  0.000000E+00  0.000000E+00  0.000000E+00  0.000000E+00  0.000000E+00  0.000000E+00  0.000000E+00  0.000000E+00       Funtag_D
      12  4.084232E+05  3.709672E+05       37456.0       36.0320     0.0182590      0.121174      0.209357      0.232250     0.0194868    0.00680543    0.00703569      0.903328       Opttag_D
      12       71.6505       2.03841       1.00000  0.000000E+00       1.00000  0.000000E+00      0.842828       39.7940       1.00000       43.7824  0.000000E+00       3.93148  0.000000E+00       3.93148  0.000000E+00  0.000000E+00      -7.86297       72.3087       2.35054        1       Scatag_D
      12       1389.92       4446.85  0.000000E+00  0.000000E+00       2115.07  0.000000E+00       2487.20       6583.29  0.000000E+00       8995.48       39.4230       11398.7 -1.000000E+10 -1.000000E+10 -1.000000E+10 -1.000000E+10 -1.000000E+10  0.000000E+00  0.000000E+00  0.000000E+00  0.000000E+00  0.000000E+00  0.000000E+00  0.000000E+00  0.000000E+00  0.000000E+00  0.000000E+00  0.000000E+00  0.000000E+00       Funtag_D
      13  4.081709E+05  3.703940E+05       37776.8       37.3808     0.0205554      0.135494      0.209035      0.232027     0.0208336    0.00746301    0.00712015      0.909388       Opttag_D
      13       71.6914       2.02494       1.00000  0.000000E+00       1.00000  0.000000E+00      0.842629       39.8387       1.00000       43.8482  0.000000E+00       3.92662  0.000000E+00       3.92662  0.000000E+00  0.000000E+00      -7.85323       72.3087       2.35054        1       Scatag_D
      13       1423.50       4506.71  0.000000E+00  0.000000E+00       2112.71  0.000000E+00       2538.28       6652.19  0.000000E+00       9075.05       39.9648       11428.4 -1.000000E+10 -1.000000E+10 -1.000000E+10 -1.000000E+10 -1.000000E+10  0.000000E+00  0.000000E+00  0.000000E+00  0.000000E+00  0.000000E+00  0.000000E+00  0.000000E+00  0.000000E+00  0.000000E+00  0.000000E+00  0.000000E+00  0.000000E+00       Funtag_D
      14  4.079714E+05  3.698629E+05       38108.4       38.6100     0.0229506      0.149345      0.208718      0.231808     0.0220816    0.00807387    0.00717394      0.915496       Opttag_D
      14       71.7385       2.01370       1.00000  0.000000E+00       1.00000  0.000000E+00      0.842442       39.8930       1.00000       43.9080  0.000000E+00       3.92252  0.000000E+00       3.92252  0.000000E+00  0.000000E+00      -7.84504       72.3087       2.35054        1       Scatag_D
      14       1445.08       4567.46  0.000000E+00  0.000000E+00       2110.49  0.000000E+00       2582.60       6745.95  0.000000E+00       9160.51       40.6348       11455.7 -1.000000E+10 -1.000000E+10 -1.000000E+10 -1.000000E+10 -1.000000E+10  0.000000E+00  0.000000E+00  0.000000E+00  0.000000E+00  0.000000E+00  0.000000E+00  0.000000E+00  0.000000E+00  0.000000E+00  0.000000E+00  0.000000E+00  0.000000E+00       Funtag_D
      15  4.077651E+05  3.696604E+05       38104.7       27.3464     0.0240351      0.154476      0.208592      0.231718     0.0225576    0.00831675    0.00715789      0.915354       Opttag_D
      15       71.7647       2.01030       1.00000  0.000000E+00       1.00000  0.000000E+00      0.842355       39.9207       1.00000       43.9332  0.000000E+00       3.92150  0.000000E+00       3.92150  0.000000E+00  0.000000E+00      -7.84300       72.3087       2.35054        1       Scatag_D
      15       1438.62       4566.03  0.000000E+00  0.000000E+00       2109.58  0.000000E+00       2591.56       6698.29  0.000000E+00       9199.74       40.9056       11460.0 -1.000000E+10 -1.000000E+10 -1.000000E+10 -1.000000E+10 -1.000000E+10  0.000000E+00  0.000000E+00  0.000000E+00  0.000000E+00  0.000000E+00  0.000000E+00  0.000000E+00  0.000000E+00  0.000000E+00  0.000000E+00  0.000000E+00  0.000000E+00       Funtag_D
      16  4.075004E+05  3.692611E+05       38239.3       29.1895     0.0263052      0.173138      0.208321      0.231542     0.0234959    0.00881808    0.00713827      0.920380       Opttag_D
      16       71.8233       2.00475       1.00000  0.000000E+00       1.00000  0.000000E+00      0.842207       39.9864       1.00000       43.9828  0.000000E+00       3.91916  0.000000E+00       3.91916  0.000000E+00  0.000000E+00      -7.83832       72.3087       2.35054        1       Scatag_D
      16       1430.75       4616.32  0.000000E+00  0.000000E+00       2107.80  0.000000E+00       2613.34       6669.86  0.000000E+00       9282.32       41.7882       11477.1 -1.000000E+10 -1.000000E+10 -1.000000E+10 -1.000000E+10 -1.000000E+10  0.000000E+00  0.000000E+00  0.000000E+00  0.000000E+00  0.000000E+00  0.000000E+00  0.000000E+00  0.000000E+00  0.000000E+00  0.000000E+00  0.000000E+00  0.000000E+00       Funtag_D
      17  4.073432E+05  3.687040E+05       38639.2       52.0541     0.0296815      0.200282      0.207940      0.231282     0.0248049    0.00949564    0.00715319      0.934993       Opttag_D
      17       71.9154       1.99843       1.00000  0.000000E+00       1.00000  0.000000E+00      0.842012       40.0936       1.00000       44.0550  0.000000E+00       3.91496  0.000000E+00       3.91496  0.000000E+00  0.000000E+00      -7.82992       72.3087       2.35054        1       Scatag_D
      17       1436.73       4764.06  0.000000E+00  0.000000E+00       2105.49  0.000000E+00       2647.46       6723.46  0.000000E+00       9405.53       43.1250       11513.3 -1.000000E+10 -1.000000E+10 -1.000000E+10 -1.000000E+10 -1.000000E+10  0.000000E+00  0.000000E+00  0.000000E+00  0.000000E+00  0.000000E+00  0.000000E+00  0.000000E+00  0.000000E+00  0.000000E+00  0.000000E+00  0.000000E+00  0.000000E+00       Funtag_D
      18  4.071394E+05  3.685000E+05       38639.4       26.8098     0.0309082      0.210094      0.207795      0.231179     0.0252844    0.00975809    0.00713672      0.929821       Opttag_D
      18       71.9519       1.99651       1.00000  0.000000E+00       1.00000  0.000000E+00      0.841944       40.1358       1.00000       44.0827  0.000000E+00       3.91297  0.000000E+00       3.91297  0.000000E+00  0.000000E+00      -7.82593       72.3087       2.35054        1       Scatag_D
      18       1430.13       4711.51  0.000000E+00  0.000000E+00       2104.70  0.000000E+00       2660.38       6710.94  0.000000E+00       9451.27       43.5693       11526.9 -1.000000E+10 -1.000000E+10 -1.000000E+10 -1.000000E+10 -1.000000E+10  0.000000E+00  0.000000E+00  0.000000E+00  0.000000E+00  0.000000E+00  0.000000E+00  0.000000E+00  0.000000E+00  0.000000E+00  0.000000E+00  0.000000E+00  0.000000E+00       Funtag_D
      19  4.069675E+05  3.681966E+05       38770.9       23.2892     0.0327278      0.224631      0.207581      0.231022     0.0259973     0.0101585    0.00715232      0.933060       Opttag_D
      19       72.0086       1.99395       1.00000  0.000000E+00       1.00000  0.000000E+00      0.841840       40.2007       1.00000       44.1255  0.000000E+00       3.90962  0.000000E+00       3.90962  0.000000E+00  0.000000E+00      -7.81923       72.3087       2.35054        1       Scatag_D
      19       1436.39       4744.39  0.000000E+00  0.000000E+00       2103.55  0.000000E+00       2678.24       6697.00  0.000000E+00       9518.52       44.1680       11548.7 -1.000000E+10 -1.000000E+10 -1.000000E+10 -1.000000E+10 -1.000000E+10  0.000000E+00  0.000000E+00  0.000000E+00  0.000000E+00  0.000000E+00  0.000000E+00  0.000000E+00  0.000000E+00  0.000000E+00  0.000000E+00  0.000000E+00  0.000000E+00       Funtag_D
      20  4.068165E+05  3.678345E+05       38982.0       27.5331     0.0349917      0.242705      0.207320      0.230833     0.0268482     0.0106358    0.00717929      0.938080       Opttag_D
      20       72.0804       1.99140       1.00000  0.000000E+00       1.00000  0.000000E+00      0.841709       40.2843       1.00000       44.1761  0.000000E+00       3.90549  0.000000E+00       3.90549  0.000000E+00  0.000000E+00      -7.81099       72.3087       2.35054        1       Scatag_D
      20       1447.24       4795.58  0.000000E+00  0.000000E+00       2102.32  0.000000E+00       2698.06       6715.30  0.000000E+00       9602.03       44.7405       11576.7 -1.000000E+10 -1.000000E+10 -1.000000E+10 -1.000000E+10 -1.000000E+10  0.000000E+00  0.000000E+00  0.000000E+00  0.000000E+00  0.000000E+00  0.000000E+00  0.000000E+00  0.000000E+00  0.000000E+00  0.000000E+00  0.000000E+00  0.000000E+00       Funtag_D
      21  4.066882E+05  3.670013E+05       39686.9       60.4917     0.0405402      0.286468      0.206714      0.230364     0.0288061     0.0117247    0.00729196      0.959372       Opttag_D
      21       72.2608       1.98740       1.00000  0.000000E+00       1.00000  0.000000E+00      0.841401       40.4948       1.00000       44.2913  0.000000E+00       3.89534  0.000000E+00       3.89534  0.000000E+00  0.000000E+00      -7.79068       72.3087       2.35054        1       Scatag_D
      21       1493.02       5015.75  0.000000E+00  0.000000E+00       2099.81  0.000000E+00       2744.27       6825.05  0.000000E+00       9809.31       45.8922       11653.8 -1.000000E+10 -1.000000E+10 -1.000000E+10 -1.000000E+10 -1.000000E+10  0.000000E+00  0.000000E+00  0.000000E+00  0.000000E+00  0.000000E+00  0.000000E+00  0.000000E+00  0.000000E+00  0.000000E+00  0.000000E+00  0.000000E+00  0.000000E+00       Funtag_D
      22  4.064487E+05  3.668270E+05       39621.8       25.0863     0.0417088      0.295763      0.206588      0.230264     0.0292157     0.0119484    0.00726024      0.949431       Opttag_D
      22       72.3011       1.98685       1.00000  0.000000E+00       1.00000  0.000000E+00      0.841326       40.5414       1.00000       44.3149  0.000000E+00       3.89306  0.000000E+00       3.89306  0.000000E+00  0.000000E+00      -7.78612       72.3087       2.35054        1       Scatag_D
      22       1480.06       4912.34  0.000000E+00  0.000000E+00       2099.47  0.000000E+00       2751.58       6814.46  0.000000E+00       9850.59       45.9338       11667.3 -1.000000E+10 -1.000000E+10 -1.000000E+10 -1.000000E+10 -1.000000E+10  0.000000E+00  0.000000E+00  0.000000E+00  0.000000E+00  0.000000E+00  0.000000E+00  0.000000E+00  0.000000E+00  0.000000E+00  0.000000E+00  0.000000E+00  0.000000E+00       Funtag_D
      23  4.063285E+05  3.667189E+05       39609.7       20.7452     0.0423701      0.301161      0.206509      0.230196     0.0294696     0.0120913    0.00725356      0.950032       Opttag_D
      23       72.3265       1.98645       1.00000  0.000000E+00       1.00000  0.000000E+00      0.841271       40.5681       1.00000       44.3272  0.000000E+00       3.89176  0.000000E+00       3.89176  0.000000E+00  0.000000E+00      -7.78352       72.3087       2.35054        1       Scatag_D
      23       1477.34       4918.55  0.000000E+00  0.000000E+00       2099.34  0.000000E+00       2752.87       6770.58  0.000000E+00       9871.63       45.7836       11673.6 -1.000000E+10 -1.000000E+10 -1.000000E+10 -1.000000E+10 -1.000000E+10  0.000000E+00  0.000000E+00  0.000000E+00  0.000000E+00  0.000000E+00  0.000000E+00  0.000000E+00  0.000000E+00  0.000000E+00  0.000000E+00  0.000000E+00  0.000000E+00       Funtag_D
      24  4.061731E+05  3.664035E+05       39769.5       26.1538     0.0445355      0.318235      0.206270      0.230001     0.0302107     0.0124871    0.00725734      0.953837       Opttag_D
      24       72.4051       1.98594       1.00000  0.000000E+00       1.00000  0.000000E+00      0.841131       40.6532       1.00000       44.3658  0.000000E+00       3.88764  0.000000E+00       3.88764  0.000000E+00  0.000000E+00      -7.77527       72.3087       2.35054        1       Scatag_D
      24       1478.88       4958.04  0.000000E+00  0.000000E+00       2098.87  0.000000E+00       2764.46       6777.22  0.000000E+00       9945.82       45.9397       11700.3 -1.000000E+10 -1.000000E+10 -1.000000E+10 -1.000000E+10 -1.000000E+10  0.000000E+00  0.000000E+00  0.000000E+00  0.000000E+00  0.000000E+00  0.000000E+00  0.000000E+00  0.000000E+00  0.000000E+00  0.000000E+00  0.000000E+00  0.000000E+00       Funtag_D
      25  4.060765E+05  3.658573E+05       40219.2       56.5376     0.0486724      0.349973      0.205839      0.229645     0.0314944     0.0131564    0.00729317      0.969580       Opttag_D
      25       72.5564       1.98664       1.00000  0.000000E+00       1.00000  0.000000E+00      0.840900       40.8121       1.00000       44.4387  0.000000E+00       3.87978  0.000000E+00       3.87978  0.000000E+00  0.000000E+00      -7.75956       72.3087       2.35054        1       Scatag_D
      25       1493.52       5123.05  0.000000E+00  0.000000E+00       2098.31  0.000000E+00       2790.55       6824.36  0.000000E+00       10088.3       46.6941       11754.4 -1.000000E+10 -1.000000E+10 -1.000000E+10 -1.000000E+10 -1.000000E+10  0.000000E+00  0.000000E+00  0.000000E+00  0.000000E+00  0.000000E+00  0.000000E+00  0.000000E+00  0.000000E+00  0.000000E+00  0.000000E+00  0.000000E+00  0.000000E+00       Funtag_D
      26  4.058899E+05  3.657392E+05       40150.7       25.7674     0.0496725      0.357491      0.205741      0.229572     0.0317717     0.0132969    0.00726479      0.960585       Opttag_D
      26       72.5939       1.98734       1.00000  0.000000E+00       1.00000  0.000000E+00      0.840852       40.8507       1.00000       44.4555  0.000000E+00       3.87777  0.000000E+00       3.87777  0.000000E+00  0.000000E+00      -7.75555       72.3087       2.35054        1       Scatag_D
      26       1481.92       5028.43  0.000000E+00  0.000000E+00       2098.32  0.000000E+00       2797.31       6812.47  0.000000E+00       10121.5       46.9066       11763.8 -1.000000E+10 -1.000000E+10 -1.000000E+10 -1.000000E+10 -1.000000E+10  0.000000E+00  0.000000E+00  0.000000E+00  0.000000E+00  0.000000E+00  0.000000E+00  0.000000E+00  0.000000E+00  0.000000E+00  0.000000E+00  0.000000E+00  0.000000E+00       Funtag_D
      27  4.057642E+05  3.655565E+05       40207.7       19.2430     0.0512727      0.369338      0.205588      0.229454     0.0322010     0.0135284    0.00726468      0.961627       Opttag_D
      27       72.6547       1.98862       1.00000  0.000000E+00       1.00000  0.000000E+00      0.840777       40.9104       1.00000       44.4846  0.000000E+00       3.87448  0.000000E+00       3.87448  0.000000E+00  0.000000E+00      -7.74895       72.3087       2.35054        1       Scatag_D
      27       1481.87       5039.35  0.000000E+00  0.000000E+00       2098.34  0.000000E+00       2808.42       6781.13  0.000000E+00       10173.4       47.2659       11777.9 -1.000000E+10 -1.000000E+10 -1.000000E+10 -1.000000E+10 -1.000000E+10  0.000000E+00  0.000000E+00  0.000000E+00  0.000000E+00  0.000000E+00  0.000000E+00  0.000000E+00  0.000000E+00  0.000000E+00  0.000000E+00  0.000000E+00  0.000000E+00       Funtag_D
      28  4.056526E+05  3.653053E+05       40347.2       23.4675     0.0536094      0.386317      0.205374      0.229276     0.0327914     0.0138804    0.00727647      0.964957       Opttag_D
      28       72.7437       1.99096       1.00000  0.000000E+00       1.00000  0.000000E+00      0.840669       40.9973       1.00000       44.5274  0.000000E+00       3.86957  0.000000E+00       3.86957  0.000000E+00  0.000000E+00      -7.73913       72.3087       2.35054        1       Scatag_D
      28       1486.69       5074.31  0.000000E+00  0.000000E+00       2098.58  0.000000E+00       2824.22       6770.29  0.000000E+00       10247.9       47.6301       11797.7 -1.000000E+10 -1.000000E+10 -1.000000E+10 -1.000000E+10 -1.000000E+10  0.000000E+00  0.000000E+00  0.000000E+00  0.000000E+00  0.000000E+00  0.000000E+00  0.000000E+00  0.000000E+00  0.000000E+00  0.000000E+00  0.000000E+00  0.000000E+00       Funtag_D
      29  4.055694E+05  3.648779E+05       40691.6       47.8600     0.0580835      0.417607      0.204997      0.228953     0.0337958     0.0145576    0.00732981      0.976919       Opttag_D
      29       72.9132       1.99686       1.00000  0.000000E+00       1.00000  0.000000E+00      0.840482       41.1588       1.00000       44.6066  0.000000E+00       3.85996  0.000000E+00       3.85996  0.000000E+00  0.000000E+00      -7.71992       72.3087       2.35054        1       Scatag_D
      29       1508.56       5200.89  0.000000E+00  0.000000E+00       2099.58  0.000000E+00       2854.15       6767.46  0.000000E+00       10384.3       47.9382       11828.7 -1.000000E+10 -1.000000E+10 -1.000000E+10 -1.000000E+10 -1.000000E+10  0.000000E+00  0.000000E+00  0.000000E+00  0.000000E+00  0.000000E+00  0.000000E+00  0.000000E+00  0.000000E+00  0.000000E+00  0.000000E+00  0.000000E+00  0.000000E+00       Funtag_D
      30  4.054133E+05  3.647641E+05       40649.2       21.1383     0.0593748      0.426287      0.204895      0.228849     0.0340631     0.0147890    0.00729695      0.970006       Opttag_D
      30       72.9613       1.99872       1.00000  0.000000E+00       1.00000  0.000000E+00      0.840430       41.2032       1.00000       44.6290  0.000000E+00       3.85720  0.000000E+00       3.85720  0.000000E+00  0.000000E+00      -7.71440       72.3087       2.35054        1       Scatag_D
      30       1495.07       5127.55  0.000000E+00  0.000000E+00       2100.04  0.000000E+00       2862.46       6761.17  0.000000E+00       10421.5       47.9428       11833.5 -1.000000E+10 -1.000000E+10 -1.000000E+10 -1.000000E+10 -1.000000E+10  0.000000E+00  0.000000E+00  0.000000E+00  0.000000E+00  0.000000E+00  0.000000E+00  0.000000E+00  0.000000E+00  0.000000E+00  0.000000E+00  0.000000E+00  0.000000E+00       Funtag_D
      31  4.053231E+05  3.646873E+05       40635.7       15.9013     0.0602968      0.431959      0.204830      0.228757     0.0342436     0.0150095    0.00729057      0.969948       Opttag_D
      31       72.9945       1.99996       1.00000  0.000000E+00       1.00000  0.000000E+00      0.840396       41.2302       1.00000       44.6430  0.000000E+00       3.85518  0.000000E+00       3.85518  0.000000E+00  0.000000E+00      -7.71037       72.3087       2.35054        1       Scatag_D
      31       1492.45       5126.94  0.000000E+00  0.000000E+00       2100.61  0.000000E+00       2867.12       6727.81  0.000000E+00       10442.3       47.7641       11830.8 -1.000000E+10 -1.000000E+10 -1.000000E+10 -1.000000E+10 -1.000000E+10  0.000000E+00  0.000000E+00  0.000000E+00  0.000000E+00  0.000000E+00  0.000000E+00  0.000000E+00  0.000000E+00  0.000000E+00  0.000000E+00  0.000000E+00  0.000000E+00       Funtag_D
      32  4.052205E+05  3.645405E+05       40680.0       20.9715     0.0621303      0.443004      0.204703      0.228586     0.0345885     0.0153931    0.00728793      0.971590       Opttag_D
      32       73.0585       2.00240       1.00000  0.000000E+00       1.00000  0.000000E+00      0.840335       41.2824       1.00000       44.6673  0.000000E+00       3.85144  0.000000E+00       3.85144  0.000000E+00  0.000000E+00      -7.70288       72.3087       2.35054        1       Scatag_D
      32       1491.37       5144.31  0.000000E+00  0.000000E+00       2101.78  0.000000E+00       2877.07       6704.36  0.000000E+00       10485.1       47.6118       11828.4 -1.000000E+10 -1.000000E+10 -1.000000E+10 -1.000000E+10 -1.000000E+10  0.000000E+00  0.000000E+00  0.000000E+00  0.000000E+00  0.000000E+00  0.000000E+00  0.000000E+00  0.000000E+00  0.000000E+00  0.000000E+00  0.000000E+00  0.000000E+00       Funtag_D
      33  4.051089E+05  3.642972E+05       40811.7       36.2734     0.0652631      0.460348      0.204491      0.228302     0.0351602     0.0159772    0.00730094      0.976936       Opttag_D
      33       73.1603       2.00584       1.00000  0.000000E+00       1.00000  0.000000E+00      0.840249       41.3595       1.00000       44.6989  0.000000E+00       3.84589  0.000000E+00       3.84589  0.000000E+00  0.000000E+00      -7.69177       72.3087       2.35054        1       Scatag_D
      33       1496.70       5201.08  0.000000E+00  0.000000E+00       2104.21  0.000000E+00       2893.37       6693.18  0.000000E+00       10550.9       47.6076       11824.6 -1.000000E+10 -1.000000E+10 -1.000000E+10 -1.000000E+10 -1.000000E+10  0.000000E+00  0.000000E+00  0.000000E+00  0.000000E+00  0.000000E+00  0.000000E+00  0.000000E+00  0.000000E+00  0.000000E+00  0.000000E+00  0.000000E+00  0.000000E+00       Funtag_D
      34  4.049783E+05  3.641249E+05       40853.4       21.6075     0.0676614      0.473050      0.204338      0.228099     0.0355651     0.0163611    0.00728036      0.973503       Opttag_D
      34       73.2357       2.00857       1.00000  0.000000E+00       1.00000  0.000000E+00      0.840188       41.4158       1.00000       44.7209  0.000000E+00       3.84213  0.000000E+00       3.84213  0.000000E+00  0.000000E+00      -7.68425       72.3087       2.35054        1       Scatag_D
      34       1488.28       5164.59  0.000000E+00  0.000000E+00       2106.28  0.000000E+00       2906.42       6712.77  0.000000E+00       10603.5       47.6208       11823.9 -1.000000E+10 -1.000000E+10 -1.000000E+10 -1.000000E+10 -1.000000E+10  0.000000E+00  0.000000E+00  0.000000E+00  0.000000E+00  0.000000E+00  0.000000E+00  0.000000E+00  0.000000E+00  0.000000E+00  0.000000E+00  0.000000E+00  0.000000E+00       Funtag_D
      35  4.048897E+05  3.640328E+05       40857.0       16.7827     0.0687012      0.477393      0.204265      0.228018     0.0357816     0.0164925    0.00727793      0.973144       Opttag_D
      35       73.2626       2.00838       1.00000  0.000000E+00       1.00000  0.000000E+00      0.840156       41.4299       1.00000       44.7257  0.000000E+00       3.84132  0.000000E+00       3.84132  0.000000E+00  0.000000E+00      -7.68264       72.3087       2.35054        1       Scatag_D
      35       1487.28       5160.78  0.000000E+00  0.000000E+00       2107.39  0.000000E+00       2910.22       6703.55  0.000000E+00       10619.0       47.6439       11821.1 -1.000000E+10 -1.000000E+10 -1.000000E+10 -1.000000E+10 -1.000000E+10  0.000000E+00  0.000000E+00  0.000000E+00  0.000000E+00  0.000000E+00  0.000000E+00  0.000000E+00  0.000000E+00  0.000000E+00  0.000000E+00  0.000000E+00  0.000000E+00       Funtag_D
      36  4.047775E+05  3.638624E+05       40915.0       20.6561     0.0705890      0.484312      0.204136      0.227878     0.0361819     0.0167170    0.00728933      0.974095       Opttag_D
      36       73.3077       2.00747       1.00000  0.000000E+00       1.00000  0.000000E+00      0.840092       41.4512       1.00000       44.7332  0.000000E+00       3.84039  0.000000E+00       3.84039  0.000000E+00  0.000000E+00      -7.68077       72.3087       2.35054        1       Scatag_D
      36       1491.94       5170.88  0.000000E+00  0.000000E+00       2109.64  0.000000E+00       2916.04       6719.19  0.000000E+00       10642.7       47.5731       11817.0 -1.000000E+10 -1.000000E+10 -1.000000E+10 -1.000000E+10 -1.000000E+10  0.000000E+00  0.000000E+00  0.000000E+00  0.000000E+00  0.000000E+00  0.000000E+00  0.000000E+00  0.000000E+00  0.000000E+00  0.000000E+00  0.000000E+00  0.000000E+00       Funtag_D
      37  4.046978E+05  3.636353E+05       41062.5       31.1345     0.0733682      0.493822      0.203962      0.227683     0.0367155     0.0170268    0.00732036      0.977223       Opttag_D
      37       73.3730       2.00685       1.00000  0.000000E+00       1.00000  0.000000E+00      0.839997       41.4869       1.00000       44.7461  0.000000E+00       3.83904  0.000000E+00       3.83904  0.000000E+00  0.000000E+00      -7.67808       72.3087       2.35054        1       Scatag_D
      37       1504.67       5204.14  0.000000E+00  0.000000E+00       2112.97  0.000000E+00       2925.15       6773.27  0.000000E+00       10678.4       47.3051       11816.6 -1.000000E+10 -1.000000E+10 -1.000000E+10 -1.000000E+10 -1.000000E+10  0.000000E+00  0.000000E+00  0.000000E+00  0.000000E+00  0.000000E+00  0.000000E+00  0.000000E+00  0.000000E+00  0.000000E+00  0.000000E+00  0.000000E+00  0.000000E+00       Funtag_D
      38  4.045982E+05  3.635664E+05       41031.8       18.1560     0.0741141      0.495311      0.203915      0.227632     0.0368775     0.0171335    0.00731104      0.974099       Opttag_D
      38       73.3864       2.00590       1.00000  0.000000E+00       1.00000  0.000000E+00      0.839962       41.4923       1.00000       44.7481  0.000000E+00       3.83923  0.000000E+00       3.83923  0.000000E+00  0.000000E+00      -7.67847       72.3087       2.35054        1       Scatag_D
      38       1500.84       5170.92  0.000000E+00  0.000000E+00       2113.94  0.000000E+00       2925.96       6775.80  0.000000E+00       10683.0       47.1055       11814.3 -1.000000E+10 -1.000000E+10 -1.000000E+10 -1.000000E+10 -1.000000E+10  0.000000E+00  0.000000E+00  0.000000E+00  0.000000E+00  0.000000E+00  0.000000E+00  0.000000E+00  0.000000E+00  0.000000E+00  0.000000E+00  0.000000E+00  0.000000E+00       Funtag_D
      39  4.045063E+05  3.634627E+05       41043.6       15.5887     0.0753128      0.497594      0.203844      0.227553     0.0371212     0.0173086    0.00731759      0.973864       Opttag_D
      39       73.4084       2.00471       1.00000  0.000000E+00       1.00000  0.000000E+00      0.839906       41.5042       1.00000       44.7523  0.000000E+00       3.83941  0.000000E+00       3.83941  0.000000E+00  0.000000E+00      -7.67882       72.3087       2.35054        1       Scatag_D
      39       1503.54       5168.42  0.000000E+00  0.000000E+00       2115.48  0.000000E+00       2927.25       6779.56  0.000000E+00       10690.9       46.8099       11811.6 -1.000000E+10 -1.000000E+10 -1.000000E+10 -1.000000E+10 -1.000000E+10  0.000000E+00  0.000000E+00  0.000000E+00  0.000000E+00  0.000000E+00  0.000000E+00  0.000000E+00  0.000000E+00  0.000000E+00  0.000000E+00  0.000000E+00  0.000000E+00       Funtag_D
      40  4.044314E+05  3.633451E+05       41086.3       17.3861     0.0768521      0.500401      0.203759      0.227448     0.0373976     0.0175224    0.00732512      0.974055       Opttag_D
      40       73.4365       2.00365       1.00000  0.000000E+00       1.00000  0.000000E+00      0.839847       41.5194       1.00000       44.7555  0.000000E+00       3.83959  0.000000E+00       3.83959  0.000000E+00  0.000000E+00      -7.67919       72.3087       2.35054        1       Scatag_D
      40       1506.63       5170.45  0.000000E+00  0.000000E+00       2117.36  0.000000E+00       2929.40       6804.32  0.000000E+00       10702.9       46.6075       11808.6 -1.000000E+10 -1.000000E+10 -1.000000E+10 -1.000000E+10 -1.000000E+10  0.000000E+00  0.000000E+00  0.000000E+00  0.000000E+00  0.000000E+00  0.000000E+00  0.000000E+00  0.000000E+00  0.000000E+00  0.000000E+00  0.000000E+00  0.000000E+00       Funtag_D
      41  4.043666E+05  3.631083E+05       41258.3       33.9410     0.0803611      0.526988      0.203590      0.227266     0.0379540     0.0178417    0.00735497      0.976419       Opttag_D
      41       73.4959       2.00158       1.00000  0.000000E+00       1.00000  0.000000E+00      0.839723       41.5508       1.00000       44.7586  0.000000E+00       3.84055  0.000000E+00       3.84055  0.000000E+00  0.000000E+00      -7.68109       72.3087       2.35054        1       Scatag_D
      41       1518.94       5195.58  0.000000E+00  0.000000E+00       2121.75  0.000000E+00       2933.71       6910.79  0.000000E+00       10729.5       46.3566       11801.6 -1.000000E+10 -1.000000E+10 -1.000000E+10 -1.000000E+10 -1.000000E+10  0.000000E+00  0.000000E+00  0.000000E+00  0.000000E+00  0.000000E+00  0.000000E+00  0.000000E+00  0.000000E+00  0.000000E+00  0.000000E+00  0.000000E+00  0.000000E+00       Funtag_D
      42  4.042596E+05  3.630696E+05       41189.9       16.3980     0.0809670      0.536219      0.203564      0.227239     0.0380448     0.0179259    0.00733986      0.974135       Opttag_D
      42       73.5031       2.00078       1.00000  0.000000E+00       1.00000  0.000000E+00      0.839701       41.5496       1.00000       44.7581  0.000000E+00       3.84107  0.000000E+00       3.84107  0.000000E+00  0.000000E+00      -7.68215       72.3087       2.35054        1       Scatag_D
      42       1512.70       5171.30  0.000000E+00  0.000000E+00       2122.37  0.000000E+00       2932.81       6876.19  0.000000E+00       10731.7       46.3638       11796.5 -1.000000E+10 -1.000000E+10 -1.000000E+10 -1.000000E+10 -1.000000E+10  0.000000E+00  0.000000E+00  0.000000E+00  0.000000E+00  0.000000E+00  0.000000E+00  0.000000E+00  0.000000E+00  0.000000E+00  0.000000E+00  0.000000E+00  0.000000E+00       Funtag_D
      43  4.041963E+05  3.630377E+05       41158.7       14.2921     0.0814505      0.544855      0.203542      0.227230     0.0381199     0.0179291    0.00733546      0.973793       Opttag_D
      43       73.5062       1.99968       1.00000  0.000000E+00       1.00000  0.000000E+00      0.839677       41.5444       1.00000       44.7563  0.000000E+00       3.84180  0.000000E+00       3.84180  0.000000E+00  0.000000E+00      -7.68360       72.3087       2.35054        1       Scatag_D
      43       1510.89       5167.67  0.000000E+00  0.000000E+00       2122.83  0.000000E+00       2930.55       6860.03  0.000000E+00       10730.9       46.4007       11789.4 -1.000000E+10 -1.000000E+10 -1.000000E+10 -1.000000E+10 -1.000000E+10  0.000000E+00  0.000000E+00  0.000000E+00  0.000000E+00  0.000000E+00  0.000000E+00  0.000000E+00  0.000000E+00  0.000000E+00  0.000000E+00  0.000000E+00  0.000000E+00       Funtag_D
      44  4.041175E+05  3.629529E+05       41164.6       16.8486     0.0829016      0.567595      0.203490      0.227208     0.0383191     0.0179314    0.00733799      0.973864       Opttag_D
      44       73.5202       1.99745       1.00000  0.000000E+00       1.00000  0.000000E+00      0.839610       41.5372       1.00000       44.7555  0.000000E+00       3.84330  0.000000E+00       3.84330  0.000000E+00  0.000000E+00      -7.68659       72.3087       2.35054        1       Scatag_D
      44       1511.93       5168.42  0.000000E+00  0.000000E+00       2124.51  0.000000E+00       2927.39       6874.88  0.000000E+00       10734.4       46.3441       11776.7 -1.000000E+10 -1.000000E+10 -1.000000E+10 -1.000000E+10 -1.000000E+10  0.000000E+00  0.000000E+00  0.000000E+00  0.000000E+00  0.000000E+00  0.000000E+00  0.000000E+00  0.000000E+00  0.000000E+00  0.000000E+00  0.000000E+00  0.000000E+00       Funtag_D
      45  4.040703E+05  3.628036E+05       41266.8       37.5509     0.0858925      0.613053      0.203399      0.227183     0.0386701     0.0178328    0.00736851      0.976603       Opttag_D
      45       73.5466       1.99336       1.00000  0.000000E+00       1.00000  0.000000E+00      0.839460       41.5198       1.00000       44.7575  0.000000E+00       3.84576  0.000000E+00       3.84576  0.000000E+00  0.000000E+00      -7.69151       72.3087       2.35054        1       Scatag_D
      45       1524.54       5197.53  0.000000E+00  0.000000E+00       2128.26  0.000000E+00       2921.75       6950.25  0.000000E+00       10743.7       46.0707       11754.7 -1.000000E+10 -1.000000E+10 -1.000000E+10 -1.000000E+10 -1.000000E+10  0.000000E+00  0.000000E+00  0.000000E+00  0.000000E+00  0.000000E+00  0.000000E+00  0.000000E+00  0.000000E+00  0.000000E+00  0.000000E+00  0.000000E+00  0.000000E+00       Funtag_D
      46  4.039645E+05  3.627654E+05       41199.1       16.5415     0.0867254      0.625091      0.203380      0.227184     0.0387598     0.0178075    0.00735080      0.973353       Opttag_D
      46       73.5536       1.99221       1.00000  0.000000E+00       1.00000  0.000000E+00      0.839418       41.5133       1.00000       44.7602  0.000000E+00       3.84646  0.000000E+00       3.84646  0.000000E+00  0.000000E+00      -7.69293       72.3087       2.35054        1       Scatag_D
      46       1517.21       5162.99  0.000000E+00  0.000000E+00       2129.37  0.000000E+00       2921.00       6927.35  0.000000E+00       10747.3       45.9885       11747.9 -1.000000E+10 -1.000000E+10 -1.000000E+10 -1.000000E+10 -1.000000E+10  0.000000E+00  0.000000E+00  0.000000E+00  0.000000E+00  0.000000E+00  0.000000E+00  0.000000E+00  0.000000E+00  0.000000E+00  0.000000E+00  0.000000E+00  0.000000E+00       Funtag_D
      47  4.039020E+05  3.627187E+05       41183.3       12.2179     0.0877234      0.639548      0.203355      0.227190     0.0388695     0.0177593    0.00734844      0.973003       Opttag_D
      47       73.5607       1.99062       1.00000  0.000000E+00       1.00000  0.000000E+00      0.839358       41.5035       1.00000       44.7654  0.000000E+00       3.84705  0.000000E+00       3.84705  0.000000E+00  0.000000E+00      -7.69409       72.3087       2.35054        1       Scatag_D
      47       1516.24       5159.28  0.000000E+00  0.000000E+00       2130.75  0.000000E+00       2919.76       6921.03  0.000000E+00       10750.9       45.8639       11739.4 -1.000000E+10 -1.000000E+10 -1.000000E+10 -1.000000E+10 -1.000000E+10  0.000000E+00  0.000000E+00  0.000000E+00  0.000000E+00  0.000000E+00  0.000000E+00  0.000000E+00  0.000000E+00  0.000000E+00  0.000000E+00  0.000000E+00  0.000000E+00       Funtag_D
      48  4.038332E+05  3.626389E+05       41194.4       14.9141     0.0895628      0.666028      0.203312      0.227202     0.0390571     0.0176691    0.00734935      0.973138       Opttag_D
      48       73.5726       1.98776       1.00000  0.000000E+00       1.00000  0.000000E+00      0.839253       41.4843       1.00000       44.7733  0.000000E+00       3.84810  0.000000E+00       3.84810  0.000000E+00  0.000000E+00      -7.69621       72.3087       2.35054        1       Scatag_D
      48       1516.62       5160.71  0.000000E+00  0.000000E+00       2133.34  0.000000E+00       2917.67       6938.23  0.000000E+00       10757.7       45.7354       11724.4 -1.000000E+10 -1.000000E+10 -1.000000E+10 -1.000000E+10 -1.000000E+10  0.000000E+00  0.000000E+00  0.000000E+00  0.000000E+00  0.000000E+00  0.000000E+00  0.000000E+00  0.000000E+00  0.000000E+00  0.000000E+00  0.000000E+00  0.000000E+00       Funtag_D
      49  4.037565E+05  3.625061E+05       41250.4       26.9734     0.0925438      0.707758      0.203241      0.227227     0.0393690     0.0175186    0.00734711      0.974544       Opttag_D
      49       73.5890       1.98266       1.00000  0.000000E+00       1.00000  0.000000E+00      0.839082       41.4494       1.00000       44.7879  0.000000E+00       3.84929  0.000000E+00       3.84929  0.000000E+00  0.000000E+00      -7.69858       72.3087       2.35054        1       Scatag_D
      49       1515.69       5175.64  0.000000E+00  0.000000E+00       2137.62  0.000000E+00       2915.48       6992.72  0.000000E+00       10768.6       45.7159       11698.8 -1.000000E+10 -1.000000E+10 -1.000000E+10 -1.000000E+10 -1.000000E+10  0.000000E+00  0.000000E+00  0.000000E+00  0.000000E+00  0.000000E+00  0.000000E+00  0.000000E+00  0.000000E+00  0.000000E+00  0.000000E+00  0.000000E+00  0.000000E+00       Funtag_D
      50  4.036657E+05  3.624123E+05       41253.4       16.2868     0.0950634      0.741838      0.203193      0.227249     0.0395894     0.0173666    0.00733579      0.973251       Opttag_D
      50       73.6019       1.97876       1.00000  0.000000E+00       1.00000  0.000000E+00      0.838949       41.4181       1.00000       44.7991  0.000000E+00       3.85060  0.000000E+00       3.85060  0.000000E+00  0.000000E+00      -7.70120       72.3087       2.35054        1       Scatag_D
      50       1511.02       5161.92  0.000000E+00  0.000000E+00       2141.48  0.000000E+00       2914.42       7021.07  0.000000E+00       10779.5       45.7458       11678.3 -1.000000E+10 -1.000000E+10 -1.000000E+10 -1.000000E+10 -1.000000E+10  0.000000E+00  0.000000E+00  0.000000E+00  0.000000E+00  0.000000E+00  0.000000E+00  0.000000E+00  0.000000E+00  0.000000E+00  0.000000E+00  0.000000E+00  0.000000E+00       Funtag_D
      51  4.035959E+05  3.623502E+05       41245.7       13.5295     0.0963603      0.758855      0.203166      0.227260     0.0397355     0.0172946    0.00732809      0.973115       Opttag_D
      51       73.6052       1.97582       1.00000  0.000000E+00       1.00000  0.000000E+00      0.838869       41.3951       1.00000       44.8056  0.000000E+00       3.85105  0.000000E+00       3.85105  0.000000E+00  0.000000E+00      -7.70210       72.3087       2.35054        1       Scatag_D
      51       1507.85       5160.48  0.000000E+00  0.000000E+00       2143.63  0.000000E+00       2913.84       7028.20  0.000000E+00       10782.8       45.7824       11663.2 -1.000000E+10 -1.000000E+10 -1.000000E+10 -1.000000E+10 -1.000000E+10  0.000000E+00  0.000000E+00  0.000000E+00  0.000000E+00  0.000000E+00  0.000000E+00  0.000000E+00  0.000000E+00  0.000000E+00  0.000000E+00  0.000000E+00  0.000000E+00       Funtag_D
      52  4.035062E+05  3.622388E+05       41267.4       18.7306     0.0985333      0.786282      0.203118      0.227279     0.0399972     0.0171475    0.00732180      0.973790       Opttag_D
      52       73.6088       1.97033       1.00000  0.000000E+00       1.00000  0.000000E+00      0.838724       41.3531       1.00000       44.8199  0.000000E+00       3.85172  0.000000E+00       3.85172  0.000000E+00  0.000000E+00      -7.70345       72.3087       2.35054        1       Scatag_D
      52       1505.27       5167.63  0.000000E+00  0.000000E+00       2147.22  0.000000E+00       2913.51       7066.58  0.000000E+00       10787.5       45.8665       11633.8 -1.000000E+10 -1.000000E+10 -1.000000E+10 -1.000000E+10 -1.000000E+10  0.000000E+00  0.000000E+00  0.000000E+00  0.000000E+00  0.000000E+00  0.000000E+00  0.000000E+00  0.000000E+00  0.000000E+00  0.000000E+00  0.000000E+00  0.000000E+00       Funtag_D
      53  4.034252E+05  3.621067E+05       41318.4       23.0162      0.101467      0.821590      0.203065      0.227317     0.0403076     0.0169238    0.00732154      0.974553       Opttag_D
      53       73.6149       1.96360       1.00000  0.000000E+00       1.00000  0.000000E+00      0.838529       41.2978       1.00000       44.8421  0.000000E+00       3.85253  0.000000E+00       3.85253  0.000000E+00  0.000000E+00      -7.70506       72.3087       2.35054        1       Scatag_D
      53       1505.16       5175.74  0.000000E+00  0.000000E+00       2152.20  0.000000E+00       2914.68       7138.33  0.000000E+00       10795.9       45.9480       11590.4 -1.000000E+10 -1.000000E+10 -1.000000E+10 -1.000000E+10 -1.000000E+10  0.000000E+00  0.000000E+00  0.000000E+00  0.000000E+00  0.000000E+00  0.000000E+00  0.000000E+00  0.000000E+00  0.000000E+00  0.000000E+00  0.000000E+00  0.000000E+00       Funtag_D
      54  4.033345E+05  3.620413E+05       41293.2       16.5474      0.102463      0.832441      0.203040      0.227325     0.0404614     0.0168676    0.00731869      0.974307       Opttag_D
      54       73.6145       1.96022       1.00000  0.000000E+00       1.00000  0.000000E+00      0.838436       41.2732       1.00000       44.8549  0.000000E+00       3.85258  0.000000E+00       3.85258  0.000000E+00  0.000000E+00      -7.70515       72.3087       2.35054        1       Scatag_D
      54       1503.99       5173.12  0.000000E+00  0.000000E+00       2153.95  0.000000E+00       2915.40       7138.04  0.000000E+00       10796.1       45.9767       11566.6 -1.000000E+10 -1.000000E+10 -1.000000E+10 -1.000000E+10 -1.000000E+10  0.000000E+00  0.000000E+00  0.000000E+00  0.000000E+00  0.000000E+00  0.000000E+00  0.000000E+00  0.000000E+00  0.000000E+00  0.000000E+00  0.000000E+00  0.000000E+00       Funtag_D
      55  4.032115E+05  3.619181E+05       41293.4       17.9279      0.104292      0.851394      0.203001      0.227339     0.0407509     0.0168051    0.00732385      0.974992       Opttag_D
      55       73.6148       1.95391       1.00000  0.000000E+00       1.00000  0.000000E+00      0.838254       41.2283       1.00000       44.8844  0.000000E+00       3.85263  0.000000E+00       3.85263  0.000000E+00  0.000000E+00      -7.70526       72.3087       2.35054        1       Scatag_D
      55       1506.11       5180.40  0.000000E+00  0.000000E+00       2156.87  0.000000E+00       2917.53       7169.08  0.000000E+00       10797.3       46.1013       11520.0 -1.000000E+10 -1.000000E+10 -1.000000E+10 -1.000000E+10 -1.000000E+10  0.000000E+00  0.000000E+00  0.000000E+00  0.000000E+00  0.000000E+00  0.000000E+00  0.000000E+00  0.000000E+00  0.000000E+00  0.000000E+00  0.000000E+00  0.000000E+00       Funtag_D
      56  4.031259E+05  3.617987E+05       41327.2       23.3511      0.106172      0.870279      0.202964      0.227339     0.0410312     0.0167895    0.00732795      0.975703       Opttag_D
      56       73.6180       1.94809       1.00000  0.000000E+00       1.00000  0.000000E+00      0.838077       41.1889       1.00000       44.9156  0.000000E+00       3.85267  0.000000E+00       3.85267  0.000000E+00  0.000000E+00      -7.70534       72.3087       2.35054        1       Scatag_D
      56       1507.80       5187.96  0.000000E+00  0.000000E+00       2159.46  0.000000E+00       2920.46       7225.77  0.000000E+00       10800.9       46.3448       11478.4 -1.000000E+10 -1.000000E+10 -1.000000E+10 -1.000000E+10 -1.000000E+10  0.000000E+00  0.000000E+00  0.000000E+00  0.000000E+00  0.000000E+00  0.000000E+00  0.000000E+00  0.000000E+00  0.000000E+00  0.000000E+00  0.000000E+00  0.000000E+00       Funtag_D
      57  4.030232E+05  3.616989E+05       41324.3       17.0642      0.107412      0.881637      0.202931      0.227323     0.0412658     0.0168900    0.00732862      0.976027       Opttag_D
      57       73.6204       1.94352       1.00000  0.000000E+00       1.00000  0.000000E+00      0.837938       41.1627       1.00000       44.9444  0.000000E+00       3.85244  0.000000E+00       3.85244  0.000000E+00  0.000000E+00      -7.70488       72.3087       2.35054        1       Scatag_D
      57       1508.07       5191.40  0.000000E+00  0.000000E+00       2160.58  0.000000E+00       2922.70       7239.30  0.000000E+00       10802.5       46.6382       11453.1 -1.000000E+10 -1.000000E+10 -1.000000E+10 -1.000000E+10 -1.000000E+10  0.000000E+00  0.000000E+00  0.000000E+00  0.000000E+00  0.000000E+00  0.000000E+00  0.000000E+00  0.000000E+00  0.000000E+00  0.000000E+00  0.000000E+00  0.000000E+00       Funtag_D
      58  4.029224E+05  3.616009E+05       41321.4       16.2007      0.108176      0.887743      0.202900      0.227276     0.0414961     0.0171294    0.00731718      0.976178       Opttag_D
      58       73.6226       1.93951       1.00000  0.000000E+00       1.00000  0.000000E+00      0.837823       41.1474       1.00000       44.9713  0.000000E+00       3.85241  0.000000E+00       3.85241  0.000000E+00  0.000000E+00      -7.70482       72.3087       2.35054        1       Scatag_D
      58       1503.37       5193.01  0.000000E+00  0.000000E+00       2160.42  0.000000E+00       2923.72       7258.07  0.000000E+00       10802.1       46.9897       11433.7 -1.000000E+10 -1.000000E+10 -1.000000E+10 -1.000000E+10 -1.000000E+10  0.000000E+00  0.000000E+00  0.000000E+00  0.000000E+00  0.000000E+00  0.000000E+00  0.000000E+00  0.000000E+00  0.000000E+00  0.000000E+00  0.000000E+00  0.000000E+00       Funtag_D
      59  4.028441E+05  3.615116E+05       41332.5       18.3303      0.108950      0.894125      0.202869      0.227225     0.0417060     0.0173487    0.00731323      0.976365       Opttag_D
      59       73.6247       1.93589       1.00000  0.000000E+00       1.00000  0.000000E+00      0.837711       41.1358       1.00000       44.9951  0.000000E+00       3.85249  0.000000E+00       3.85249  0.000000E+00  0.000000E+00      -7.70498       72.3087       2.35054        1       Scatag_D
      59       1501.74       5195.00  0.000000E+00  0.000000E+00       2159.72  0.000000E+00       2924.69       7282.27  0.000000E+00       10801.7       47.2518       11420.1 -1.000000E+10 -1.000000E+10 -1.000000E+10 -1.000000E+10 -1.000000E+10  0.000000E+00  0.000000E+00  0.000000E+00  0.000000E+00  0.000000E+00  0.000000E+00  0.000000E+00  0.000000E+00  0.000000E+00  0.000000E+00  0.000000E+00  0.000000E+00       Funtag_D
      60  4.027714E+05  3.614484E+05       41323.0       14.6362      0.109497      0.898776      0.202847      0.227181     0.0418544     0.0175204    0.00731493      0.976216       Opttag_D
      60       73.6258       1.93336       1.00000  0.000000E+00       1.00000  0.000000E+00      0.837626       41.1292       1.00000       45.0096  0.000000E+00       3.85266  0.000000E+00       3.85266  0.000000E+00  0.000000E+00      -7.70532       72.3087       2.35054        1       Scatag_D
      60       1502.44       5193.41  0.000000E+00  0.000000E+00       2158.66  0.000000E+00       2925.20       7283.59  0.000000E+00       10800.8       47.3856       11411.5 -1.000000E+10 -1.000000E+10 -1.000000E+10 -1.000000E+10 -1.000000E+10  0.000000E+00  0.000000E+00  0.000000E+00  0.000000E+00  0.000000E+00  0.000000E+00  0.000000E+00  0.000000E+00  0.000000E+00  0.000000E+00  0.000000E+00  0.000000E+00       Funtag_D
      61  4.026912E+05  3.613493E+05       41341.9       14.6623      0.110526      0.908120      0.202819      0.227127     0.0420874     0.0177443    0.00732347      0.976188       Opttag_D
      61       73.6267       1.92899       1.00000  0.000000E+00       1.00000  0.000000E+00      0.837472       41.1124       1.00000       45.0350  0.000000E+00       3.85326  0.000000E+00       3.85326  0.000000E+00  0.000000E+00      -7.70652       72.3087       2.35054        1       Scatag_D
      61       1505.95       5193.12  0.000000E+00  0.000000E+00       2156.48  0.000000E+00       2926.37       7310.96  0.000000E+00       10799.5       47.5000       11402.0 -1.000000E+10 -1.000000E+10 -1.000000E+10 -1.000000E+10 -1.000000E+10  0.000000E+00  0.000000E+00  0.000000E+00  0.000000E+00  0.000000E+00  0.000000E+00  0.000000E+00  0.000000E+00  0.000000E+00  0.000000E+00  0.000000E+00  0.000000E+00       Funtag_D
      62  4.026318E+05  3.612689E+05       41362.9       17.1470      0.111546      0.917616      0.202794      0.227097     0.0422763     0.0178753    0.00732925      0.976406       Opttag_D
      62       73.6282       1.92526       1.00000  0.000000E+00       1.00000  0.000000E+00      0.837323       41.0946       1.00000       45.0576  0.000000E+00       3.85368  0.000000E+00       3.85368  0.000000E+00  0.000000E+00      -7.70736       72.3087       2.35054        1       Scatag_D
      62       1508.33       5195.43  0.000000E+00  0.000000E+00       2154.52  0.000000E+00       2928.08       7331.96  0.000000E+00       10798.3       47.5444       11398.7 -1.000000E+10 -1.000000E+10 -1.000000E+10 -1.000000E+10 -1.000000E+10  0.000000E+00  0.000000E+00  0.000000E+00  0.000000E+00  0.000000E+00  0.000000E+00  0.000000E+00  0.000000E+00  0.000000E+00  0.000000E+00  0.000000E+00  0.000000E+00       Funtag_D
      63  4.025700E+05  3.612172E+05       41352.8       13.2413      0.112225      0.931296      0.202782      0.227084     0.0423977     0.0179528    0.00732709      0.976250       Opttag_D
      63       73.6289       1.92274       1.00000  0.000000E+00       1.00000  0.000000E+00      0.837215       41.0814       1.00000       45.0740  0.000000E+00       3.85402  0.000000E+00       3.85402  0.000000E+00  0.000000E+00      -7.70804       72.3087       2.35054        1       Scatag_D
      63       1507.44       5193.78  0.000000E+00  0.000000E+00       2153.04  0.000000E+00       2929.26       7329.96  0.000000E+00       10796.7       47.5279       11395.1 -1.000000E+10 -1.000000E+10 -1.000000E+10 -1.000000E+10 -1.000000E+10  0.000000E+00  0.000000E+00  0.000000E+00  0.000000E+00  0.000000E+00  0.000000E+00  0.000000E+00  0.000000E+00  0.000000E+00  0.000000E+00  0.000000E+00  0.000000E+00       Funtag_D
      64  4.024753E+05  3.610903E+05       41385.1       14.7381      0.113927      0.969362      0.202750      0.227055     0.0426960     0.0181388    0.00732480      0.976688       Opttag_D
      64       73.6313       1.91636       1.00000  0.000000E+00       1.00000  0.000000E+00      0.836947       41.0442       1.00000       45.1160  0.000000E+00       3.85508  0.000000E+00       3.85508  0.000000E+00  0.000000E+00      -7.71016       72.3087       2.35054        1       Scatag_D
      64       1506.50       5198.44  0.000000E+00  0.000000E+00       2149.43  0.000000E+00       2933.16       7367.49  0.000000E+00       10793.0       47.5535       11389.5 -1.000000E+10 -1.000000E+10 -1.000000E+10 -1.000000E+10 -1.000000E+10  0.000000E+00  0.000000E+00  0.000000E+00  0.000000E+00  0.000000E+00  0.000000E+00  0.000000E+00  0.000000E+00  0.000000E+00  0.000000E+00  0.000000E+00  0.000000E+00       Funtag_D
      65  4.024146E+05  3.609689E+05       41445.7       22.6008      0.115583       1.00105      0.202719      0.227004     0.0429812     0.0183679    0.00732327      0.977752       Opttag_D
      65       73.6341       1.91030       1.00000  0.000000E+00       1.00000  0.000000E+00      0.836696       41.0092       1.00000       45.1577  0.000000E+00       3.85631  0.000000E+00       3.85631  0.000000E+00  0.000000E+00      -7.71263       72.3087       2.35054        1       Scatag_D
      65       1505.87       5209.77  0.000000E+00  0.000000E+00       2146.05  0.000000E+00       2937.50       7421.31  0.000000E+00       10789.8       47.7123       11387.6 -1.000000E+10 -1.000000E+10 -1.000000E+10 -1.000000E+10 -1.000000E+10  0.000000E+00  0.000000E+00  0.000000E+00  0.000000E+00  0.000000E+00  0.000000E+00  0.000000E+00  0.000000E+00  0.000000E+00  0.000000E+00  0.000000E+00  0.000000E+00       Funtag_D
      66  4.023437E+05  3.609211E+05       41422.7       15.3485      0.116169       1.00403      0.202706      0.226983     0.0430937     0.0184774    0.00731828      0.977602       Opttag_D
      66       73.6346       1.90782       1.00000  0.000000E+00       1.00000  0.000000E+00      0.836597       40.9942       1.00000       45.1724  0.000000E+00       3.85683  0.000000E+00       3.85683  0.000000E+00  0.000000E+00      -7.71365       72.3087       2.35054        1       Scatag_D
      66       1503.82       5208.17  0.000000E+00  0.000000E+00       2144.50  0.000000E+00       2939.03       7410.51  0.000000E+00       10787.0       47.8015       11381.8 -1.000000E+10 -1.000000E+10 -1.000000E+10 -1.000000E+10 -1.000000E+10  0.000000E+00  0.000000E+00  0.000000E+00  0.000000E+00  0.000000E+00  0.000000E+00  0.000000E+00  0.000000E+00  0.000000E+00  0.000000E+00  0.000000E+00  0.000000E+00       Funtag_D
      67  4.022492E+05  3.608114E+05       41437.9       12.4656      0.117578       1.01025      0.202681      0.226931     0.0433514     0.0187334    0.00731900      0.977707       Opttag_D
      67       73.6353       1.90191       1.00000  0.000000E+00       1.00000  0.000000E+00      0.836369       40.9546       1.00000       45.2074  0.000000E+00       3.85829  0.000000E+00       3.85829  0.000000E+00  0.000000E+00      -7.71658       72.3087       2.35054        1       Scatag_D
      67       1504.11       5209.29  0.000000E+00  0.000000E+00       2140.96  0.000000E+00       2942.62       7440.85  0.000000E+00       10780.5       48.0896       11371.4 -1.000000E+10 -1.000000E+10 -1.000000E+10 -1.000000E+10 -1.000000E+10  0.000000E+00  0.000000E+00  0.000000E+00  0.000000E+00  0.000000E+00  0.000000E+00  0.000000E+00  0.000000E+00  0.000000E+00  0.000000E+00  0.000000E+00  0.000000E+00       Funtag_D
      68  4.021936E+05  3.607276E+05       41466.0       15.1298      0.118839       1.02085      0.202661      0.226894     0.0435483     0.0189170    0.00732397      0.977921       Opttag_D
      68       73.6366       1.89724       1.00000  0.000000E+00       1.00000  0.000000E+00      0.836171       40.9214       1.00000       45.2390  0.000000E+00       3.85915  0.000000E+00       3.85915  0.000000E+00  0.000000E+00      -7.71829       72.3087       2.35054        1       Scatag_D
      68       1506.16       5211.57  0.000000E+00  0.000000E+00       2138.45  0.000000E+00       2945.46       7473.38  0.000000E+00       10775.6       48.3272       11367.1 -1.000000E+10 -1.000000E+10 -1.000000E+10 -1.000000E+10 -1.000000E+10  0.000000E+00  0.000000E+00  0.000000E+00  0.000000E+00  0.000000E+00  0.000000E+00  0.000000E+00  0.000000E+00  0.000000E+00  0.000000E+00  0.000000E+00  0.000000E+00       Funtag_D
      69  4.021091E+05  3.606094E+05       41499.7       13.7938      0.120779       1.03400      0.202639      0.226843     0.0438259     0.0191886    0.00734110      0.978192       Opttag_D
      69       73.6390       1.89029       1.00000  0.000000E+00       1.00000  0.000000E+00      0.835872       40.8663       1.00000       45.2894  0.000000E+00       3.86049  0.000000E+00       3.86049  0.000000E+00  0.000000E+00      -7.72099       72.3087       2.35054        1       Scatag_D
      69       1513.21       5214.46  0.000000E+00  0.000000E+00       2135.22  0.000000E+00       2949.53       7510.77  0.000000E+00       10766.9       48.7039       11360.9 -1.000000E+10 -1.000000E+10 -1.000000E+10 -1.000000E+10 -1.000000E+10  0.000000E+00  0.000000E+00  0.000000E+00  0.000000E+00  0.000000E+00  0.000000E+00  0.000000E+00  0.000000E+00  0.000000E+00  0.000000E+00  0.000000E+00  0.000000E+00       Funtag_D
      70  4.020452E+05  3.605010E+05       41544.2       25.1591      0.122755       1.04661      0.202620      0.226828     0.0440806     0.0193570    0.00735174      0.978834       Opttag_D
      70       73.6441       1.88353       1.00000  0.000000E+00       1.00000  0.000000E+00      0.835562       40.8035       1.00000       45.3446  0.000000E+00       3.86164  0.000000E+00       3.86164  0.000000E+00  0.000000E+00      -7.72328       72.3087       2.35054        1       Scatag_D
      70       1517.60       5221.30  0.000000E+00  0.000000E+00       2133.09  0.000000E+00       2953.05       7572.97  0.000000E+00       10753.8       48.9286       11343.5 -1.000000E+10 -1.000000E+10 -1.000000E+10 -1.000000E+10 -1.000000E+10  0.000000E+00  0.000000E+00  0.000000E+00  0.000000E+00  0.000000E+00  0.000000E+00  0.000000E+00  0.000000E+00  0.000000E+00  0.000000E+00  0.000000E+00  0.000000E+00       Funtag_D
      71  4.019619E+05  3.604739E+05       41488.0       13.0432      0.123265       1.05074      0.202612      0.226830     0.0441444     0.0194099    0.00734365      0.978291       Opttag_D
      71       73.6471       1.88202       1.00000  0.000000E+00       1.00000  0.000000E+00      0.835477       40.7882       1.00000       45.3600  0.000000E+00       3.86152  0.000000E+00       3.86152  0.000000E+00  0.000000E+00      -7.72304       72.3087       2.35054        1       Scatag_D
      71       1514.26       5215.51  0.000000E+00  0.000000E+00       2132.79  0.000000E+00       2953.32       7544.72  0.000000E+00       10747.5       48.8912       11331.1 -1.000000E+10 -1.000000E+10 -1.000000E+10 -1.000000E+10 -1.000000E+10  0.000000E+00  0.000000E+00  0.000000E+00  0.000000E+00  0.000000E+00  0.000000E+00  0.000000E+00  0.000000E+00  0.000000E+00  0.000000E+00  0.000000E+00  0.000000E+00       Funtag_D
      72  4.018991E+05  3.604635E+05       41435.6       11.1741      0.123396       1.05291      0.202606      0.226845     0.0441690     0.0194011    0.00732799      0.977798       Opttag_D
      72       73.6505       1.88165       1.00000  0.000000E+00       1.00000  0.000000E+00      0.835443       40.7813       1.00000       45.3649  0.000000E+00       3.86106  0.000000E+00       3.86106  0.000000E+00  0.000000E+00      -7.72212       72.3087       2.35054        1       Scatag_D
      72       1507.81       5210.26  0.000000E+00  0.000000E+00       2132.73  0.000000E+00       2952.21       7532.70  0.000000E+00       10738.7       48.6864       11312.5 -1.000000E+10 -1.000000E+10 -1.000000E+10 -1.000000E+10 -1.000000E+10  0.000000E+00  0.000000E+00  0.000000E+00  0.000000E+00  0.000000E+00  0.000000E+00  0.000000E+00  0.000000E+00  0.000000E+00  0.000000E+00  0.000000E+00  0.000000E+00       Funtag_D
      73  4.018415E+05  3.604352E+05       41406.3       13.0147      0.123978       1.06213      0.202595      0.226855     0.0442353     0.0194331    0.00731863      0.977707       Opttag_D
      73       73.6566       1.88028       1.00000  0.000000E+00       1.00000  0.000000E+00      0.835353       40.7627       1.00000       45.3784  0.000000E+00       3.86084  0.000000E+00       3.86084  0.000000E+00  0.000000E+00      -7.72169       72.3087       2.35054        1       Scatag_D
      73       1503.96       5209.29  0.000000E+00  0.000000E+00       2132.88  0.000000E+00       2951.55       7537.49  0.000000E+00       10727.6       48.4665       11295.0 -1.000000E+10 -1.000000E+10 -1.000000E+10 -1.000000E+10 -1.000000E+10  0.000000E+00  0.000000E+00  0.000000E+00  0.000000E+00  0.000000E+00  0.000000E+00  0.000000E+00  0.000000E+00  0.000000E+00  0.000000E+00  0.000000E+00  0.000000E+00       Funtag_D
      74  4.018032E+05  3.603848E+05       41418.4       24.1466      0.125276       1.08404      0.202572      0.226872     0.0443539     0.0194782    0.00732056      0.978443       Opttag_D
      74       73.6713       1.87803       1.00000  0.000000E+00       1.00000  0.000000E+00      0.835177       40.7290       1.00000       45.4059  0.000000E+00       3.86029  0.000000E+00       3.86029  0.000000E+00  0.000000E+00      -7.72058       72.3087       2.35054        1       Scatag_D
      74       1504.76       5217.14  0.000000E+00  0.000000E+00       2133.97  0.000000E+00       2950.04       7576.11  0.000000E+00       10709.7       48.0327       11278.6 -1.000000E+10 -1.000000E+10 -1.000000E+10 -1.000000E+10 -1.000000E+10  0.000000E+00  0.000000E+00  0.000000E+00  0.000000E+00  0.000000E+00  0.000000E+00  0.000000E+00  0.000000E+00  0.000000E+00  0.000000E+00  0.000000E+00  0.000000E+00       Funtag_D
      75  4.017389E+05  3.603688E+05       41370.1       9.98393      0.125477       1.08721      0.202566      0.226857     0.0443913     0.0195331    0.00731865      0.977935       Opttag_D
      75       73.6734       1.87741       1.00000  0.000000E+00       1.00000  0.000000E+00      0.835151       40.7232       1.00000       45.4106  0.000000E+00       3.86043  0.000000E+00       3.86043  0.000000E+00  0.000000E+00      -7.72087       72.3087       2.35054        1       Scatag_D
      75       1503.97       5211.72  0.000000E+00  0.000000E+00       2134.08  0.000000E+00       2948.91       7542.51  0.000000E+00       10707.7       48.0139       11273.2 -1.000000E+10 -1.000000E+10 -1.000000E+10 -1.000000E+10 -1.000000E+10  0.000000E+00  0.000000E+00  0.000000E+00  0.000000E+00  0.000000E+00  0.000000E+00  0.000000E+00  0.000000E+00  0.000000E+00  0.000000E+00  0.000000E+00  0.000000E+00       Funtag_D
      76  4.017079E+05  3.603478E+05       41360.1       9.07170      0.125774       1.09129      0.202559      0.226841     0.0444407     0.0195874    0.00732146      0.977696       Opttag_D
      76       73.6770       1.87671       1.00000  0.000000E+00       1.00000  0.000000E+00      0.835113       40.7165       1.00000       45.4174  0.000000E+00       3.86050  0.000000E+00       3.86050  0.000000E+00  0.000000E+00      -7.72100       72.3087       2.35054        1       Scatag_D
      76       1505.13       5209.18  0.000000E+00  0.000000E+00       2134.24  0.000000E+00       2947.16       7546.64  0.000000E+00       10704.4       47.9883       11265.4 -1.000000E+10 -1.000000E+10 -1.000000E+10 -1.000000E+10 -1.000000E+10  0.000000E+00  0.000000E+00  0.000000E+00  0.000000E+00  0.000000E+00  0.000000E+00  0.000000E+00  0.000000E+00  0.000000E+00  0.000000E+00  0.000000E+00  0.000000E+00       Funtag_D
      77  4.016683E+05  3.603119E+05       41356.4       11.1381      0.126393       1.09815      0.202544      0.226816     0.0445252     0.0196793    0.00732790      0.977423       Opttag_D
      77       73.6863       1.87567       1.00000  0.000000E+00       1.00000  0.000000E+00      0.835031       40.7038       1.00000       45.4321  0.000000E+00       3.86031  0.000000E+00       3.86031  0.000000E+00  0.000000E+00      -7.72062       72.3087       2.35054        1       Scatag_D
      77       1507.78       5206.26  0.000000E+00  0.000000E+00       2134.69  0.000000E+00       2943.87       7568.56  0.000000E+00       10696.1       47.9007       11251.2 -1.000000E+10 -1.000000E+10 -1.000000E+10 -1.000000E+10 -1.000000E+10  0.000000E+00  0.000000E+00  0.000000E+00  0.000000E+00  0.000000E+00  0.000000E+00  0.000000E+00  0.000000E+00  0.000000E+00  0.000000E+00  0.000000E+00  0.000000E+00       Funtag_D
      78  4.016180E+05  3.602419E+05       41376.2       26.4576      0.128003       1.11041      0.202516      0.226760     0.0446896     0.0198478    0.00734298      0.976937       Opttag_D
      78       73.7150       1.87419       1.00000  0.000000E+00       1.00000  0.000000E+00      0.834831       40.6744       1.00000       45.4687  0.000000E+00       3.85953  0.000000E+00       3.85953  0.000000E+00  0.000000E+00      -7.71906       72.3087       2.35054        1       Scatag_D
      78       1513.99       5201.09  0.000000E+00  0.000000E+00       2135.87  0.000000E+00       2935.74       7655.13  0.000000E+00       10673.6       47.7025       11213.1 -1.000000E+10 -1.000000E+10 -1.000000E+10 -1.000000E+10 -1.000000E+10  0.000000E+00  0.000000E+00  0.000000E+00  0.000000E+00  0.000000E+00  0.000000E+00  0.000000E+00  0.000000E+00  0.000000E+00  0.000000E+00  0.000000E+00  0.000000E+00       Funtag_D
      79  4.015387E+05  3.602162E+05       41322.5       13.6591      0.128927       1.11655      0.202504      0.226728     0.0447500     0.0199612    0.00734793      0.976454       Opttag_D
      79       73.7325       1.87393       1.00000  0.000000E+00       1.00000  0.000000E+00      0.834723       40.6600       1.00000       45.4895  0.000000E+00       3.85905  0.000000E+00       3.85905  0.000000E+00  0.000000E+00      -7.71810       72.3087       2.35054        1       Scatag_D
      79       1516.03       5195.94  0.000000E+00  0.000000E+00       2136.68  0.000000E+00       2931.95       7638.87  0.000000E+00       10661.8       47.5566       11193.7 -1.000000E+10 -1.000000E+10 -1.000000E+10 -1.000000E+10 -1.000000E+10  0.000000E+00  0.000000E+00  0.000000E+00  0.000000E+00  0.000000E+00  0.000000E+00  0.000000E+00  0.000000E+00  0.000000E+00  0.000000E+00  0.000000E+00  0.000000E+00       Funtag_D
      80  4.014802E+05  3.602097E+05       41270.5       10.2791      0.129726       1.11854      0.202495      0.226703     0.0447653     0.0200471    0.00734039      0.976001       Opttag_D
      80       73.7506       1.87457       1.00000  0.000000E+00       1.00000  0.000000E+00      0.834639       40.6508       1.00000       45.5052  0.000000E+00       3.85842  0.000000E+00       3.85842  0.000000E+00  0.000000E+00      -7.71684       72.3087       2.35054        1       Scatag_D
      80       1512.92       5191.13  0.000000E+00  0.000000E+00       2137.26  0.000000E+00       2928.46       7631.59  0.000000E+00       10650.4       47.3771       11171.4 -1.000000E+10 -1.000000E+10 -1.000000E+10 -1.000000E+10 -1.000000E+10  0.000000E+00  0.000000E+00  0.000000E+00  0.000000E+00  0.000000E+00  0.000000E+00  0.000000E+00  0.000000E+00  0.000000E+00  0.000000E+00  0.000000E+00  0.000000E+00       Funtag_D
      81  4.014308E+05  3.602152E+05       41215.6       10.9514      0.130650       1.11986      0.202494      0.226677     0.0447523     0.0201493    0.00732929      0.975554       Opttag_D
      81       73.7724       1.87587       1.00000  0.000000E+00       1.00000  0.000000E+00      0.834558       40.6420       1.00000       45.5198  0.000000E+00       3.85785  0.000000E+00       3.85785  0.000000E+00  0.000000E+00      -7.71570       72.3087       2.35054        1       Scatag_D
      81       1508.35       5186.37  0.000000E+00  0.000000E+00       2137.74  0.000000E+00       2924.46       7629.71  0.000000E+00       10637.8       47.1064       11144.1 -1.000000E+10 -1.000000E+10 -1.000000E+10 -1.000000E+10 -1.000000E+10  0.000000E+00  0.000000E+00  0.000000E+00  0.000000E+00  0.000000E+00  0.000000E+00  0.000000E+00  0.000000E+00  0.000000E+00  0.000000E+00  0.000000E+00  0.000000E+00       Funtag_D
      82  4.014112E+05  3.602369E+05       41174.2       27.9716      0.132116       1.12176      0.202498      0.226623     0.0447013     0.0203592    0.00731478      0.975117       Opttag_D
      82       73.8086       1.87877       1.00000  0.000000E+00       1.00000  0.000000E+00      0.834456       40.6352       1.00000       45.5377  0.000000E+00       3.85674  0.000000E+00       3.85674  0.000000E+00  0.000000E+00      -7.71349       72.3087       2.35054        1       Scatag_D
      82       1502.38       5181.73  0.000000E+00  0.000000E+00       2138.04  0.000000E+00       2917.29       7664.38  0.000000E+00       10619.8       46.5241       11104.1 -1.000000E+10 -1.000000E+10 -1.000000E+10 -1.000000E+10 -1.000000E+10  0.000000E+00  0.000000E+00  0.000000E+00  0.000000E+00  0.000000E+00  0.000000E+00  0.000000E+00  0.000000E+00  0.000000E+00  0.000000E+00  0.000000E+00  0.000000E+00       Funtag_D
      83  4.013434E+05  3.602421E+05       41101.4       10.3090      0.132171       1.12058      0.202498      0.226623     0.0446892     0.0203855    0.00730689      0.974621       Opttag_D
      83       73.8110       1.87914       1.00000  0.000000E+00       1.00000  0.000000E+00      0.834460       40.6359       1.00000       45.5365  0.000000E+00       3.85687  0.000000E+00       3.85687  0.000000E+00  0.000000E+00      -7.71373       72.3087       2.35054        1       Scatag_D
      83       1499.14       5176.46  0.000000E+00  0.000000E+00       2137.78  0.000000E+00       2916.57       7608.20  0.000000E+00       10618.2       46.4656       11098.6 -1.000000E+10 -1.000000E+10 -1.000000E+10 -1.000000E+10 -1.000000E+10  0.000000E+00  0.000000E+00  0.000000E+00  0.000000E+00  0.000000E+00  0.000000E+00  0.000000E+00  0.000000E+00  0.000000E+00  0.000000E+00  0.000000E+00  0.000000E+00       Funtag_D
      84  4.013132E+05  3.602443E+05       41068.9       7.45525      0.132165       1.11925      0.202497      0.226620     0.0446839     0.0204291    0.00730082      0.974298       Opttag_D
      84       73.8127       1.87949       1.00000  0.000000E+00       1.00000  0.000000E+00      0.834472       40.6391       1.00000       45.5333  0.000000E+00       3.85694  0.000000E+00       3.85694  0.000000E+00  0.000000E+00      -7.71388       72.3087       2.35054        1       Scatag_D
      84       1496.65       5173.03  0.000000E+00  0.000000E+00       2137.26  0.000000E+00       2915.46       7592.24  0.000000E+00       10617.0       46.3923       11090.8 -1.000000E+10 -1.000000E+10 -1.000000E+10 -1.000000E+10 -1.000000E+10  0.000000E+00  0.000000E+00  0.000000E+00  0.000000E+00  0.000000E+00  0.000000E+00  0.000000E+00  0.000000E+00  0.000000E+00  0.000000E+00  0.000000E+00  0.000000E+00       Funtag_D
      85  4.012798E+05  3.602471E+05       41032.8       9.99010      0.132527       1.11763      0.202494      0.226605     0.0446775     0.0205339    0.00729588      0.973899       Opttag_D
      85       73.8244       1.88054       1.00000  0.000000E+00       1.00000  0.000000E+00      0.834456       40.6425       1.00000       45.5358  0.000000E+00       3.85668  0.000000E+00       3.85668  0.000000E+00  0.000000E+00      -7.71335       72.3087       2.35054        1       Scatag_D
      85       1494.63       5168.79  0.000000E+00  0.000000E+00       2136.64  0.000000E+00       2912.24       7586.43  0.000000E+00       10611.5       46.2447       11076.3 -1.000000E+10 -1.000000E+10 -1.000000E+10 -1.000000E+10 -1.000000E+10  0.000000E+00  0.000000E+00  0.000000E+00  0.000000E+00  0.000000E+00  0.000000E+00  0.000000E+00  0.000000E+00  0.000000E+00  0.000000E+00  0.000000E+00  0.000000E+00       Funtag_D
      86  4.012233E+05  3.602354E+05       40987.9       14.9597      0.133147       1.11321      0.202480      0.226594     0.0447050     0.0206650    0.00729250      0.973327       Opttag_D
      86       73.8458       1.88194       1.00000  0.000000E+00       1.00000  0.000000E+00      0.834408       40.6503       1.00000       45.5458  0.000000E+00       3.85594  0.000000E+00       3.85594  0.000000E+00  0.000000E+00      -7.71188       72.3087       2.35054        1       Scatag_D
      86       1493.24       5162.72  0.000000E+00  0.000000E+00       2135.48  0.000000E+00       2906.65       7586.35  0.000000E+00       10601.8       46.2406       11055.4 -1.000000E+10 -1.000000E+10 -1.000000E+10 -1.000000E+10 -1.000000E+10  0.000000E+00  0.000000E+00  0.000000E+00  0.000000E+00  0.000000E+00  0.000000E+00  0.000000E+00  0.000000E+00  0.000000E+00  0.000000E+00  0.000000E+00  0.000000E+00       Funtag_D
      87  4.011881E+05  3.602318E+05       40956.3       19.4704      0.134506       1.10707      0.202464      0.226564     0.0447133     0.0208882    0.00729944      0.972875       Opttag_D
      87       73.8873       1.88506       1.00000  0.000000E+00       1.00000  0.000000E+00      0.834313       40.6616       1.00000       45.5654  0.000000E+00       3.85491  0.000000E+00       3.85491  0.000000E+00  0.000000E+00      -7.70983       72.3087       2.35054        1       Scatag_D
      87       1496.09       5157.93  0.000000E+00  0.000000E+00       2134.47  0.000000E+00       2898.67       7599.34  0.000000E+00       10586.0       46.1844       11037.6 -1.000000E+10 -1.000000E+10 -1.000000E+10 -1.000000E+10 -1.000000E+10  0.000000E+00  0.000000E+00  0.000000E+00  0.000000E+00  0.000000E+00  0.000000E+00  0.000000E+00  0.000000E+00  0.000000E+00  0.000000E+00  0.000000E+00  0.000000E+00       Funtag_D
      88  4.011428E+05  3.602227E+05       40920.1       8.75127      0.134459       1.10699      0.202462      0.226578     0.0447347     0.0208367    0.00729403      0.972644       Opttag_D
      88       73.8862       1.88467       1.00000  0.000000E+00       1.00000  0.000000E+00      0.834302       40.6614       1.00000       45.5674  0.000000E+00       3.85489  0.000000E+00       3.85489  0.000000E+00  0.000000E+00      -7.70977       72.3087       2.35054        1       Scatag_D
      88       1493.87       5155.48  0.000000E+00  0.000000E+00       2134.49  0.000000E+00       2898.75       7573.68  0.000000E+00       10585.5       46.2240       11032.1 -1.000000E+10 -1.000000E+10 -1.000000E+10 -1.000000E+10 -1.000000E+10  0.000000E+00  0.000000E+00  0.000000E+00  0.000000E+00  0.000000E+00  0.000000E+00  0.000000E+00  0.000000E+00  0.000000E+00  0.000000E+00  0.000000E+00  0.000000E+00       Funtag_D
      89  4.011167E+05  3.602146E+05       40902.1       8.11317      0.134570       1.10645      0.202457      0.226587     0.0447538     0.0208062    0.00728891      0.972497       Opttag_D
      89       73.8897       1.88464       1.00000  0.000000E+00       1.00000  0.000000E+00      0.834280       40.6623       1.00000       45.5711  0.000000E+00       3.85484  0.000000E+00       3.85484  0.000000E+00  0.000000E+00      -7.70968       72.3087       2.35054        1       Scatag_D
      89       1491.77       5153.92  0.000000E+00  0.000000E+00       2134.50  0.000000E+00       2898.27       7569.67  0.000000E+00       10583.2       46.2233       11024.6 -1.000000E+10 -1.000000E+10 -1.000000E+10 -1.000000E+10 -1.000000E+10  0.000000E+00  0.000000E+00  0.000000E+00  0.000000E+00  0.000000E+00  0.000000E+00  0.000000E+00  0.000000E+00  0.000000E+00  0.000000E+00  0.000000E+00  0.000000E+00       Funtag_D
      90  4.010845E+05  3.602078E+05       40876.7       10.0838      0.135009       1.10486      0.202448      0.226597     0.0447697     0.0207966    0.00728448      0.972359       Opttag_D
      90       73.9026       1.88538       1.00000  0.000000E+00       1.00000  0.000000E+00      0.834236       40.6658       1.00000       45.5787  0.000000E+00       3.85473  0.000000E+00       3.85473  0.000000E+00  0.000000E+00      -7.70946       72.3087       2.35054        1       Scatag_D
      90       1489.96       5152.46  0.000000E+00  0.000000E+00       2134.48  0.000000E+00       2896.52       7567.08  0.000000E+00       10577.4       46.1242       11012.7 -1.000000E+10 -1.000000E+10 -1.000000E+10 -1.000000E+10 -1.000000E+10  0.000000E+00  0.000000E+00  0.000000E+00  0.000000E+00  0.000000E+00  0.000000E+00  0.000000E+00  0.000000E+00  0.000000E+00  0.000000E+00  0.000000E+00  0.000000E+00       Funtag_D
      91  4.010628E+05  3.602064E+05       40856.4       25.1745      0.136452       1.15449      0.202434      0.226598     0.0447729     0.0208546    0.00728474      0.972367       Opttag_D
      91       73.9448       1.88855       1.00000  0.000000E+00       1.00000  0.000000E+00      0.834126       40.6778       1.00000       45.5991  0.000000E+00       3.85412  0.000000E+00       3.85412  0.000000E+00  0.000000E+00      -7.70824       72.3087       2.35054        1       Scatag_D
      91       1490.07       5152.54  0.000000E+00  0.000000E+00       2134.80  0.000000E+00       2890.38       7588.37  0.000000E+00       10563.9       45.6694       10990.7 -1.000000E+10 -1.000000E+10 -1.000000E+10 -1.000000E+10 -1.000000E+10  0.000000E+00  0.000000E+00  0.000000E+00  0.000000E+00  0.000000E+00  0.000000E+00  0.000000E+00  0.000000E+00  0.000000E+00  0.000000E+00  0.000000E+00  0.000000E+00       Funtag_D
      92  4.010099E+05  3.602088E+05       40801.1       10.2448      0.136732       1.16695      0.202431      0.226604     0.0447674     0.0208465    0.00728425      0.972327       Opttag_D
      92       73.9524       1.88918       1.00000  0.000000E+00       1.00000  0.000000E+00      0.834105       40.6804       1.00000       45.6026  0.000000E+00       3.85401  0.000000E+00       3.85401  0.000000E+00  0.000000E+00      -7.70803       72.3087       2.35054        1       Scatag_D
      92       1489.87       5152.11  0.000000E+00  0.000000E+00       2134.97  0.000000E+00       2889.39       7540.39  0.000000E+00       10561.8       45.5470       10987.1 -1.000000E+10 -1.000000E+10 -1.000000E+10 -1.000000E+10 -1.000000E+10  0.000000E+00  0.000000E+00  0.000000E+00  0.000000E+00  0.000000E+00  0.000000E+00  0.000000E+00  0.000000E+00  0.000000E+00  0.000000E+00  0.000000E+00  0.000000E+00       Funtag_D
      93  4.009890E+05  3.602130E+05       40776.0       6.77143      0.137013       1.17977      0.202430      0.226601     0.0447575     0.0208633    0.00728357      0.972239       Opttag_D
      93       73.9600       1.88989       1.00000  0.000000E+00       1.00000  0.000000E+00      0.834089       40.6824       1.00000       45.6057  0.000000E+00       3.85387  0.000000E+00       3.85387  0.000000E+00  0.000000E+00      -7.70774       72.3087       2.35054        1       Scatag_D
      93       1489.59       5151.19  0.000000E+00  0.000000E+00       2135.12  0.000000E+00       2887.75       7525.03  0.000000E+00       10559.9       45.4083       10982.1 -1.000000E+10 -1.000000E+10 -1.000000E+10 -1.000000E+10 -1.000000E+10  0.000000E+00  0.000000E+00  0.000000E+00  0.000000E+00  0.000000E+00  0.000000E+00  0.000000E+00  0.000000E+00  0.000000E+00  0.000000E+00  0.000000E+00  0.000000E+00       Funtag_D
      94  4.009677E+05  3.602192E+05       40748.5       7.68260      0.137573       1.20435      0.202431      0.226600     0.0447429     0.0208800    0.00728455      0.972185       Opttag_D
      94       73.9750       1.89118       1.00000  0.000000E+00       1.00000  0.000000E+00      0.834053       40.6858       1.00000       45.6133  0.000000E+00       3.85356  0.000000E+00       3.85356  0.000000E+00  0.000000E+00      -7.70711       72.3087       2.35054        1       Scatag_D
      94       1489.99       5150.61  0.000000E+00  0.000000E+00       2135.47  0.000000E+00       2884.91       7512.17  0.000000E+00       10556.4       45.2220       10973.7 -1.000000E+10 -1.000000E+10 -1.000000E+10 -1.000000E+10 -1.000000E+10  0.000000E+00  0.000000E+00  0.000000E+00  0.000000E+00  0.000000E+00  0.000000E+00  0.000000E+00  0.000000E+00  0.000000E+00  0.000000E+00  0.000000E+00  0.000000E+00       Funtag_D
      95  4.009311E+05  3.602268E+05       40704.2       9.79365      0.138415       1.23852      0.202429      0.226604     0.0447249     0.0208654    0.00728340      0.972074       Opttag_D
      95       73.9960       1.89279       1.00000  0.000000E+00       1.00000  0.000000E+00      0.833991       40.6880       1.00000       45.6272  0.000000E+00       3.85315  0.000000E+00       3.85315  0.000000E+00  0.000000E+00      -7.70631       72.3087       2.35054        1       Scatag_D
      95       1489.52       5149.44  0.000000E+00  0.000000E+00       2136.07  0.000000E+00       2881.45       7491.49  0.000000E+00       10551.6       45.1181       10959.6 -1.000000E+10 -1.000000E+10 -1.000000E+10 -1.000000E+10 -1.000000E+10  0.000000E+00  0.000000E+00  0.000000E+00  0.000000E+00  0.000000E+00  0.000000E+00  0.000000E+00  0.000000E+00  0.000000E+00  0.000000E+00  0.000000E+00  0.000000E+00       Funtag_D
      96  4.009230E+05  3.602529E+05       40670.1       25.6049      0.140694       1.32441      0.202430      0.226621     0.0446636     0.0207881    0.00729022      0.972586       Opttag_D
      96       74.0481       1.89662       1.00000  0.000000E+00       1.00000  0.000000E+00      0.833832       40.6864       1.00000       45.6667  0.000000E+00       3.85268  0.000000E+00       3.85268  0.000000E+00  0.000000E+00      -7.70535       72.3087       2.35054        1       Scatag_D
      96       1492.31       5154.86  0.000000E+00  0.000000E+00       2138.30  0.000000E+00       2875.55       7484.15  0.000000E+00       10543.7       45.0179       10936.2 -1.000000E+10 -1.000000E+10 -1.000000E+10 -1.000000E+10 -1.000000E+10  0.000000E+00  0.000000E+00  0.000000E+00  0.000000E+00  0.000000E+00  0.000000E+00  0.000000E+00  0.000000E+00  0.000000E+00  0.000000E+00  0.000000E+00  0.000000E+00       Funtag_D
      97  4.008601E+05  3.602441E+05       40616.0       9.00644      0.140507       1.31558      0.202428      0.226626     0.0446844     0.0207610    0.00728169      0.972190       Opttag_D
      97       74.0425       1.89593       1.00000  0.000000E+00       1.00000  0.000000E+00      0.833836       40.6848       1.00000       45.6653  0.000000E+00       3.85278  0.000000E+00       3.85278  0.000000E+00  0.000000E+00      -7.70557       72.3087       2.35054        1       Scatag_D
      97       1488.82       5150.67  0.000000E+00  0.000000E+00       2137.94  0.000000E+00       2876.73       7438.64  0.000000E+00       10543.7       45.1423       10934.4 -1.000000E+10 -1.000000E+10 -1.000000E+10 -1.000000E+10 -1.000000E+10  0.000000E+00  0.000000E+00  0.000000E+00  0.000000E+00  0.000000E+00  0.000000E+00  0.000000E+00  0.000000E+00  0.000000E+00  0.000000E+00  0.000000E+00  0.000000E+00       Funtag_D
      98  4.008406E+05  3.602367E+05       40603.9       7.29007      0.140477       1.31238      0.202427      0.226628     0.0447019     0.0207424    0.00727553      0.972098       Opttag_D
      98       74.0401       1.89539       1.00000  0.000000E+00       1.00000  0.000000E+00      0.833830       40.6818       1.00000       45.6665  0.000000E+00       3.85296  0.000000E+00       3.85296  0.000000E+00  0.000000E+00      -7.70593       72.3087       2.35054        1       Scatag_D
      98       1486.30       5149.69  0.000000E+00  0.000000E+00       2137.71  0.000000E+00       2877.85       7432.14  0.000000E+00       10543.2       45.2549       10931.7 -1.000000E+10 -1.000000E+10 -1.000000E+10 -1.000000E+10 -1.000000E+10  0.000000E+00  0.000000E+00  0.000000E+00  0.000000E+00  0.000000E+00  0.000000E+00  0.000000E+00  0.000000E+00  0.000000E+00  0.000000E+00  0.000000E+00  0.000000E+00       Funtag_D
      99  4.008066E+05  3.602268E+05       40579.9       8.80565      0.141000       1.32674      0.202425      0.226637     0.0447251     0.0206855    0.00726742      0.972126       Opttag_D
      99       74.0469       1.89512       1.00000  0.000000E+00       1.00000  0.000000E+00      0.833782       40.6750       1.00000       45.6775  0.000000E+00       3.85332  0.000000E+00       3.85332  0.000000E+00  0.000000E+00      -7.70664       72.3087       2.35054        1       Scatag_D
      99       1482.99       5149.99  0.000000E+00  0.000000E+00       2137.80  0.000000E+00       2879.51       7418.81  0.000000E+00       10540.9       45.4189       10924.5 -1.000000E+10 -1.000000E+10 -1.000000E+10 -1.000000E+10 -1.000000E+10  0.000000E+00  0.000000E+00  0.000000E+00  0.000000E+00  0.000000E+00  0.000000E+00  0.000000E+00  0.000000E+00  0.000000E+00  0.000000E+00  0.000000E+00  0.000000E+00       Funtag_D

   MaxLik Scaling Cycle 0105

   Wilson plot

   Statistics in resolution bins after refinement cycle 0003
```


|  |  |
| --- | --- |
|  | - Rxpct factors plot- Rxpct factors table- <Log-Lik Gain>- Ln Sigma A- Correlation Coefficients |


```
   Iters     Tot_Funct    Xray_Funct    Geom_Funct          Grms     rmsD2init     maxD2init             R         Rfree           LLG       LLGfree     rms(bond)    rms(angle)
   Iters             K   B_IMPF_FRAG        K_MISS   B_TEMP_MISS   K_IMPF_MISS   B_IMPF_MISS        K_SOLV   B_TEMP_SOLV   K_IMPF_SOLV   B_IMPF_SOLV        B_TEMP     B_TEMP_11     B_TEMP_12     B_TEMP_22     B_TEMP_13     B_TEMP_23     B_TEMP_33      K_WILSON      B_WILSON      FGCYCLES
   Iters          BOND         ANGLE      IMPROPER       TORSION    SINTORSION      TRIGONAL        PLANAR  BCORRELATION       CONTACT     IDEALDIST        CHIRAL    SIMILARITY       OCCSSUM  UTILDISTANCE     UTILANGLE   UTILTORSION        RHOFIT        ADP-01        ADP-02        ADP-03        ADP-04        ADP-05        ADP-06        ADP-07        ADP-08        ADP-09        ADP-10      RHOFITPC            QM
     100  4.007832E+05  3.602135E+05       40569.7       21.5944      0.142468       1.37178      0.202420      0.226634     0.0447564     0.0206580    0.00726840      0.972513       Opttag_D
     100       74.0698       1.89563       1.00000  0.000000E+00       1.00000  0.000000E+00      0.833676       40.6651       1.00000       45.7024  0.000000E+00       3.85361  0.000000E+00       3.85361  0.000000E+00  0.000000E+00      -7.70721       72.3087       2.35054        1       Scatag_D
     100       1483.39       5154.09  0.000000E+00  0.000000E+00       2138.49  0.000000E+00       2881.17       7416.61  0.000000E+00       10536.0       45.3913       10914.6 -1.000000E+10 -1.000000E+10 -1.000000E+10 -1.000000E+10 -1.000000E+10  0.000000E+00  0.000000E+00  0.000000E+00  0.000000E+00  0.000000E+00  0.000000E+00  0.000000E+00  0.000000E+00  0.000000E+00  0.000000E+00  0.000000E+00  0.000000E+00       Funtag_D
   Geometry Restraint Deviations SCREEN Information

 
 No -bcorrel_zeroocchydrogens_factor flag found - keeping default: will set B for OCC=0 hydrogens to    1.200 times heavy-atom B


 TLS information. Total number of groups:     2


 Group   Natoms    FixRB   FixTLS   Group Tag    Group Spec
     1     2768      Y        Y     tls1    { A|* }
     2     2770      Y        Y     tls2    { B|* }
 
NOTE BUSTER_TLS_SET tls1 { A|* }
NOTE BUSTER_TLS_T   tls1     -6.89402E-02  9.73396E-02  1.12143E-02 -5.14314E-02  3.70007E-02 -6.09601E-02
NOTE BUSTER_TLS_L   tls1      2.34061E+00  1.26720E+00  1.13030E+00 -7.76197E-01 -1.07598E+00  5.55382E-01
NOTE BUSTER_TLS_S   tls1      2.71047E-02 -3.50758E-01  5.52083E-01 -3.65020E-01  2.02419E-01 -2.02943E-01  6.79091E-03 -2.19839E-01
NOTE BUSTER_TLS_O   tls1     -2.32263E+01 -8.41675E+01  1.09752E+01
NOTE BUSTER_TLS_FIX tls1 ALL
 
NOTE BUSTER_TLS_SET tls2 { B|* }
NOTE BUSTER_TLS_T   tls2     -1.17934E-01 -4.34381E-02  3.08544E-02  1.57672E-02  3.71279E-02 -2.24165E-02
NOTE BUSTER_TLS_L   tls2      1.51304E+00  9.47989E-01  1.07060E+00  6.77124E-01 -9.34394E-01 -7.04506E-01
NOTE BUSTER_TLS_S   tls2     -4.36251E-03  8.11357E-02 -1.53271E-01  1.51330E-01  1.63309E-01  5.90040E-02 -7.47994E-02  3.57312E-02
NOTE BUSTER_TLS_O   tls2      2.22079E+01 -7.23190E+01  3.56420E+00
NOTE BUSTER_TLS_FIX tls2 ALL

 For final position, Geometry function summary
     term                           number     weight       rms        GooF      function     fn/numb
     ================================================================================================
     BOND bond lengths (angs)       5653       2.000      0.0073       0.362      1483.39       0.262
     ANGL bond angles (degs)        7670       2.000        0.97       0.580      5154.09       0.672
     TORS split-harmonic tors       1270       0.000       16.70       1.051         0.00       0.000
     SINTOR sinusoidal tors         1270       2.000       16.70                  2138.49       1.684
     PLAN general plane (angs)       963       5.000      0.0155       0.774      2881.17       2.992
     BCORrelations (angs^2)         5653      10.000       1.015       0.362      7416.61       1.312
     CONT (bad contacts)               0       5.000                                 0.00
     IDEAL(ideal-dist contact)      5151       4.000                             10535.96
     CHIRAL (gelly semiharmon)       740       5.000                                45.39
     SIM similarity restraint for NCS and/or TARGET                              10914.60
     OCCSUM restraint on sum of occupanies                                           0.00       as none defined
     DISTAN utility restraints interatomic distance                                  0.00       as none defined
     UTILANGLE utility angle restraints                                              0.00       as none defined
     UTILTOR utility restraints on torsion angles                                    0.00       as none defined
     Peptide omega torsion angles                           3.27 degs - monitor only
 
 WARNING: Have |delta/sigma| deviations > 5.0 sigma. Number of outliers for each term: 
 WARNING:         1 bond lengths. Worst is     5.3 sigs     1.48 Angs A|104:SD=CE (MET)
 WARNING:         1 idealD contacts. Worst     5.0 sigs     2.61 Angs A|104:CE(MET)=S|459:O(HOH) symm: 1555=5555

 Weighted rms      0.0073         0.973  (temporary rms bond and angle for BUSTER to pick up!)
 Weighted intra-residual rms(bond) for StdProtein (excluding H)      0.0071
 Weighted intra-residual rms(bond) for Buffer     (excluding H)      0.0030
 Outputing geometry progress graph in plotmtv format to file:Geom_Res.mtv

 ############### Running final geometry/screen analysis (screen_final.txt)

 s/r gelly normal termination
```

Statistics and scale factors vs. cycle number
```

|  |  |  |
| --- | --- | --- |
|  | - Rxpct factors- <Log-Lik Gain>- GEOM Residuals- Ln Sigma_A_ | - Scale Factors- B Factors- Imperfection Factors |

```
   Final Geometry Information


---


### 2. Final results

Statistics and scale factors vs. cycle number
```

|  |  |  |
| --- | --- | --- |
|  | - Rxpct factors- <Log-Lik Gain>- GEOM Residuals- Ln Sigma_A_ | - Scale Factors- B Factors- Imperfection Factors |

```
   Average Figure of Merit
   Structure Factor Amplitudes


 # normal termination

  Program stopped.
```

closesok message: time spent in tree = 1877.60 (user) 40.44 (sys)

---

# Normal termination

```
 Resources usage summary:
     Time spent in client ...................  1877.60 (user)       40.46 (system)
     Time spent in scripts (    27 calls) ...     3.52 (user)        0.18 (system)
     Time spent in server ...................     0.01 (user)        0.00 (system)

     Max resident memory used (kb) .......... 467704
     Number of page faults .................. 0 (major)    1014851 (minor)
     Number of context switches ............. 537008 (niced)    7632 (other)
```
